# Bile acid metabolism and signaling in human and animal health: A One Health perspective

**DOI:** 10.1002/imo2.70123

**Published:** 2026-07-17

**Authors:** Lei Li, Shuai Liu, Zhijun Cao, Yangchun Cao, Shengru Wu, Lu Deng, Junhu Yao, Jun Zhang

**Affiliations:** ^1^ College of Animal Science and Technology Northwest A&F University Yangling China; ^2^ State Key Laboratory of Animal Nutrition and Feeding, International Calf and Heifer Organization, College of Animal Science and Technology China Agricultural University Beijing China

**Keywords:** bile acids, bile acid metabolism, gut microbiota, bile acid signaling, human health, animal health, One Health

## Abstract

The One Health concept emphasizes the interdependence of human, animal, and ecosystem health. Against this broad backdrop, bile acids (BAs), as central products of host‐microbiota co‐metabolism, act far beyond their classical role in lipid emulsification. This review systematically summarizes BA biosynthesis, enterohepatic circulation, and gut microbiota‐mediated metabolic modification. We highlight how gut microbiota‐derived secondary BAs and farnesoid X receptor and Takeda G protein‐coupled receptor 5‐mediated BA signaling together shape BA homeostasis, glucose and lipid metabolism, intestinal barrier integrity, and immune homeostasis. We further discuss how population diversity, circadian rhythm, diet, behavior, environmental exposure, and surgical interventions dynamically shape systemic BA profiles. Finally, from a One Health perspective, we highlight the translational potential of BA‐targeted strategies in hepatobiliary diseases, metabolic disorders, neurodegenerative and retinal degenerative diseases, and intestinal infections, as well as their value in animal production as non‐antibiotic nutritional regulators. By improving animal health and reducing excessive drug use, residues, and risks of antimicrobial resistance at the source, BA‐based precision nutrition may contribute to food safety and ecosystem health. Overall, this review positions BAs as key molecular hubs in One Health, linking host metabolism, gut microbiota, immune regulation, and the integrated health of humans, animals, and ecosystems.

## Highlights

- Bile acids (BAs) act as host‐microbiota co‐metabolic hubs linking human, animal, and ecosystem health.
- BAs regulate glucose and lipid metabolism, intestinal barrier, and immune homeostasis through receptor signaling and microbiota‐derived metabolites.
- BAs are dynamically influenced by population diversity, circadian rhythm, diet, behavior, environmental exposure, and surgery.
- BAs provide therapeutic and nutritional strategies for human disease intervention and animal health, further supporting food safety, sustainable development, and One Health benefits.

## Graphical abstract

Bile acids maintain host homeostasis through enterohepatic circulation, microbial transformation, and bile acid receptor signaling, thereby regulating glucose and lipid metabolism, intestinal barrier integrity, and immunity. These effects support human disease intervention and health‐oriented animal production, reducing drug dependence and residue risks while promoting food safety, sustainability, and One Health benefits.

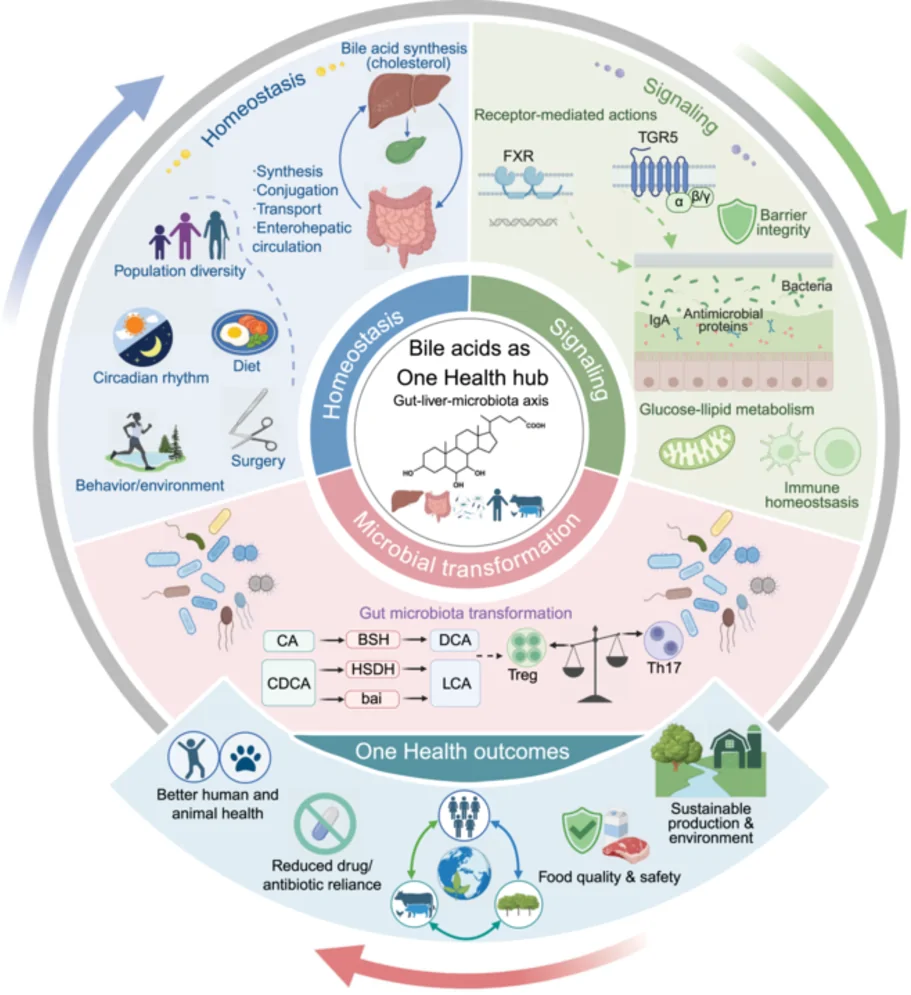

## 1 INTRODUCTION

Metabolic disorders have become a shared challenge affecting both human public health and the sustainable development of modern animal production [1, 2]. In human health, the burden of chronic liver and metabolic diseases, such as cholestatic liver diseases and metabolic dysfunction‐associated steatotic liver disease (MASLD), continues to increase [3, 4]. The onset and progression of these diseases are jointly influenced by metabolic homeostasis, dietary habits, gut microbiota, and environmental exposures, thereby imposing growing pressure on healthcare resources and chronic disease prevention and control. In intensive animal production, metabolic dysfunction, impaired intestinal barrier integrity, immune imbalance, and reduced production performance are also increasingly prominent, further increasing reliance on pharmacological interventions and aggravating food safety risks and environmental emission pressures [5, 6]. These problems not only compromise human and animal health but may also create cascading pressures among public health, food safety, and ecosystems through increased dependence on antibiotics and other drugs, the risk of antimicrobial resistance, manure discharge, and reduced resource‐use efficiency.

Addressing this complex challenge requires an integrated framework that links the health of humans, animals, and the broader ecosystems they inhabit. In 2021, the One Health High‐Level Expert Panel defined One Health as an approach that recognizes the interconnection and interdependence of human, animal, and ecosystem health [7]. Therefore, within the One Health framework, identifying key molecular nodes that coordinate metabolism, gut microbiota, and immune homeostasis across species is of great significance for understanding and intervening in metabolic disorders. Bile acids (BAs), as important products of host‐gut microbiota co‐metabolism, are increasingly recognized as key molecular nodes connecting human, animal, and ecosystem health [8]. Within the enterohepatic circulation, BAs are converted into diverse secondary or modified BA species and transported throughout the host. By acting on nuclear receptors, such as the farnesoid X receptor (FXR), and membrane receptors, such as the Takeda G protein‐coupled receptor 5 (TGR5), BAs regulate glucose and lipid metabolism and influence intestinal barrier integrity, inflammatory responses, and immune homeostasis [9].

Therefore, from a One Health perspective, we first outline BA biosynthesis, enterohepatic circulation, gut microbiota‐mediated transformation, and extrahepatic metabolism. We then systematically summarize the mechanisms by which BA receptors regulate metabolic homeostasis, intestinal barrier integrity, and immune homeostasis, and further analyze how population differences, circadian rhythm, dietary habits, behavioral and environmental factors, and surgical interventions shape BA metabolism. On this basis, we synthesize recent advances in the application of BAs in human diseases and animal health production, with particular emphasis on their potential value for BA‐targeted therapy, precision nutritional regulation, reduced drug dependence, and sustainable livestock production. Overall, this review provides a One Health‐oriented framework for understanding how BA‐centered mechanisms and interventions may support disease intervention, precision nutrition, reduced drug dependence, and healthier, more efficient animal production.

## 2 BA SYNTHESIS AND METABOLISM

### BA biosynthesis

BAs are amphipathic cholesterol‐derived metabolites synthesized mainly in hepatocytes, and their production represents a major route for cholesterol catabolism and elimination in humans [10]. BA biosynthesis proceeds through two well‐recognized pathways: the classical (neutral) pathway and the alternative (acidic) pathway (Figure 1) [10]. The classical pathway initiates with the hydroxylation of cholesterol to 7α‐hydroxycholesterol, mediated by cholesterol 7α‐hydroxylase (CYP7A1) in hepatocytes. As the rate‐limiting enzyme, CYP7A1 determines the overall yield and synthesis rate. 7α‐hydroxycholesterol is converted to 7α‐hydroxy‐4‐cholesten‐3‐one by 3β‐hydroxysteroid dehydrogenase 7. Subsequently, the metabolism of the intermediate 7α‐hydroxy‐4‐cholesten‐3‐one diverges. Under the catalysis of sterol 12α‐hydroxylase (CYP8B1), the pathway proceeds toward cholic acid (CA). Conversely, in the absence of CYP8B1 activity, chenodeoxycholic acid (CDCA) is formed via side‐chain oxidation mediated by sterol 27‐hydroxylase (CYP27A1). Notably, the final products in both branches rely on CYP27A1‐mediated side‐chain oxidation [11, 12]. The alternative pathway initiates with side‐chain oxidation mediated by CYP27A1, followed by catalysis by oxysterol 7α‐hydroxylase, ultimately leading to the synthesis of CDCA [13]. Although BAs synthesized via the alternative pathway account for a minor proportion (approximately 10%) of the total pool in humans, this pathway serves as a critical compensatory mechanism, which is significantly upregulated when the classical pathway is inhibited [14]. For instance, under pathological conditions such as hepatic disease, the alternative pathway may become the predominant route for BA synthesis [15]. In rodent hepatocytes (e.g., mice), the alternative pathway is more active, accounting for approximately 25% of total BA synthesis. Uniquely, rodents possess the enzyme sterol 6β‐hydroxylase, which efficiently converts CDCA into α‐muricholic acid and β‐muricholic acid (β‐MCA). Additionally, sterol 6β‐hydroxylase can metabolize ursodeoxycholic acid (UDCA) into β‐MCA [16, 17]. In pig hepatocytes, CDCA is primarily metabolized into hyocholic acid via 6α‐hydroxylation, a species‐specific trait that distinguishes porcine BA composition from that of humans and rodents [18].

**Figure 1:**
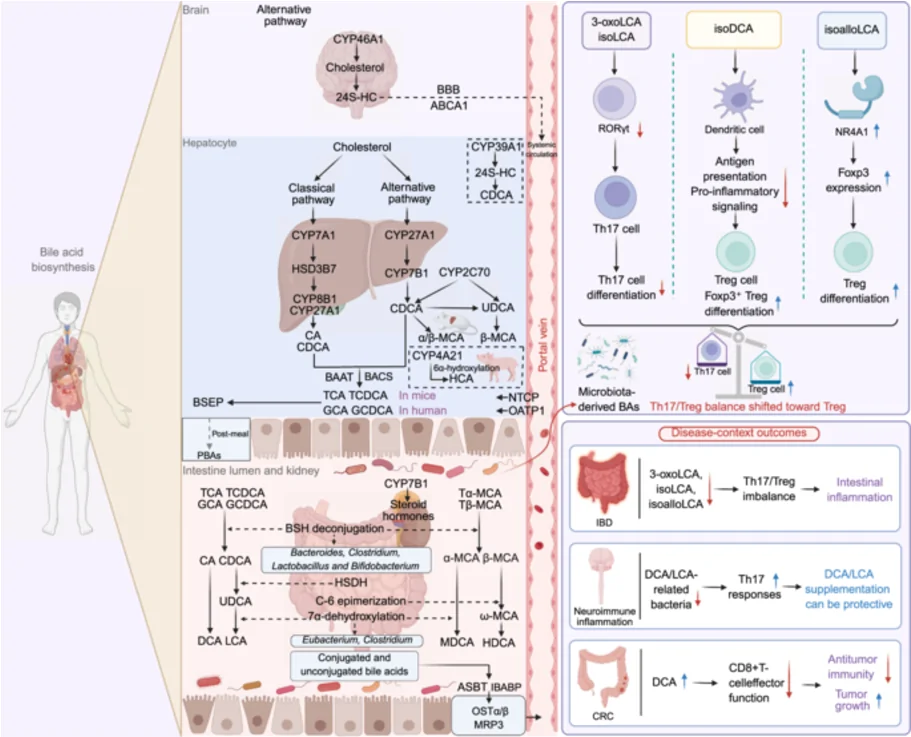
BA synthesis, circulation, microbial transformation, and T cell immune regulation. Cholesterol is converted into cholic acid (CA) and chenodeoxycholic acid (CDCA) through the classical or alternative pathways in hepatocytes, followed by conjugation and secretion into the intestine, where bile acids (BAs) facilitate lipid absorption and undergo enterohepatic circulation. A small fraction of BAs reaches the colon and is transformed by microbial enzymes, including bile salt hydrolase (BSH), hydroxysteroid dehydrogenase (HSDH), and 7α‐dehydroxylases, into deoxycholic acid (DCA), lithocholic acid (LCA), and related derivatives. These metabolites regulate the balance between T helper 17 (Th17) cells and regulatory T (Treg) cells: 3‐oxoLCA and isoLCA suppress Th17 differentiation, whereas isoDCA and isoalloLCA promote Treg generation. Their immunological effects depend on BA species, local concentration, target cell type, and disease context. Blue arrows represent an increase, and red arrows represent a decrease. 24S‐HC, 24S‐hydroxycholesterol; ABCA1, ATP‐binding cassette transporter A1; ASBT, apical sodium‐dependent bile acid transporter; BAAT, bile acid‐CoA:amino acid *N*‐acyltransferase; BACS, bile acid‐CoA synthetase; BBB, blood–brain barrier; BSEP, bile salt export pump; CD8^+^ T cell, cluster of differentiation 8‐positive T cellCRC, colorectal cancer; CYP27A1, sterol 27‐hydroxylase; CYP2C70, sterol 6β‐hydroxylase; CYP39A1, 24‐hydroxycholesterol 7α‐hydroxylase; CYP46A1, cholesterol 24‐hydroxylase; CYP4A21, cytochrome P450 family 4 subfamily A member 21; CYP7A1, cholesterol 7α‐hydroxylase; CYP7B1, oxysterol 7α‐hydroxylase; CYP8B1, sterol 12α‐hydroxylase; GCA, glycocholic acid; GCDCA, glycochenodeoxycholic acid; Foxp3, forkhead box P3; HCA, hyocholic acid; HDCA, hyodeoxycholic acid; HSD3B7, 3β‐hydroxysteroid dehydrogenase 7; IBABP, ileal bile acid‐binding protein; IBD, inflammatory bowel disease; MCA, muricholic acid; α‐MCA, α‐muricholic acid; β‐MCA, β‐muricholic acid; Tα‐MCA, tauro‐α‐muricholic acid; Tβ‐MCA, tauro‐β‐muricholic acid; ω‐MCA, ω‐muricholic acid; MDCA, murideoxycholic acid; MRP3, multidrug resistance‐associated protein 3; NR4A1, nuclear receptor subfamily 4 group A member 1; NTCP, sodium‐taurocholate cotransporting polypeptide; OATP1, organic anion‐transporting polypeptide 1; OSTα/β, organic solute transporter α/β; PBA, primary bile acid; RORγt, retinoic acid receptor‐related orphan receptor γt; TCA, taurocholic acid; TCDCA, taurochenodeoxycholic acid; UDCA, ursodeoxycholic acid.

### Enterohepatic circulation

Primary bile acids (PBAs), including CA and CDCA, are conjugated with taurine, predominantly in mice, or glycine, predominantly in humans. This two‐step process first involves the activation of BAs to CoA thioesters by BA‐CoA synthetase, followed by amide bond formation catalyzed by BA‐CoA:amino acid N‐acyltransferase. The resulting conjugated bile acids (CBAs) are secreted from hepatocytes into the bile canaliculus via the bile salt export pump (BSEP) [19]. In parallel, sulfated or glucuronidated BAs are excreted into bile via multidrug resistance‐associated protein 2 (MRP2) [19]. Conjugation markedly lowers the pKa of BAs, increasing their ionization at physiological pH, reducing passive membrane permeability, and thereby limiting cytotoxicity [20].

After meals, gallbladder contraction releases bile containing BAs, cholesterol, and phospholipids into the intestinal lumen, where BAs facilitate lipid emulsification, micelle formation, and lipid absorption. Intestinal transporters then ensure efficient BA reclamation to the liver through the portal circulation. Although a small fraction of BAs, particularly unconjugated bile acids, can be passively absorbed along the intestine, approximately 95% of BAs are actively reabsorbed in the distal ileum via the apical sodium‐dependent bile acid transporter (ASBT) [21]. By contrast, sulfated or glucuronidated BAs are poorly reabsorbed and are largely eliminated [22]. After entering enterocytes through ASBT, BAs bind to ileal bile acid‐binding protein, which facilitates their intracellular transport to the basolateral membrane. They are then exported into the portal circulation via organic solute transporter α/β and MRP3 [10]. After returning to the liver, BAs are taken up by hepatocytes through the sodium‐taurocholate cotransporting polypeptide (NTCP) and organic anion‐transporting polypeptides, and are subsequently resecreted into the bile canaliculus by BSEP together with newly synthesized BAs. This cyclical process is termed enterohepatic circulation [23]. In humans, BAs undergo approximately 4–12 enterohepatic cycles per day, while the liver synthesizes 0.2–0.6 g of new BAs to maintain a relatively constant pool of approximately 3 g [24]. Under conditions of cholestasis or BA overload, basolateral efflux transporters, such as MRP3 and MRP4, may promote BA export into the systemic circulation, facilitating alternative elimination, including renal excretion [25, 26].

### Intestinal microbial metabolism

The gut microbiota functions as a key metabolic organ that markedly diversifies the BA pool. Approximately 5% of BAs escape ileal reabsorption and enter the colon, where gut microbes extensively remodel BA composition. A major initial microbial reaction is the deconjugation of CBAs to unconjugated PBAs, catalyzed by bile salt hydrolases (BSH), which are widely distributed among genera such as *Bacteroides*, *Clostridium*, *Lactobacillus*, and *Bifidobacterium* [27]. In the anaerobic colonic environment, another critical transformation is 7α‐dehydroxylation, a process mediated by enzymes encoded by the *bai* operon, which is enriched in *Clostridium cluster XIVa*. This reaction converts CA to deoxycholic acid (DCA) and CDCA to lithocholic acid (LCA), generating the major secondary bile acids (SBAs) [27]. In addition, hydroxysteroid dehydrogenases (HSDH) catalyze oxidation, reduction, and epimerization reactions that further expand BA structural diversity. For example, CDCA can be converted to UDCA through HSDH‐mediated epimerization, and UDCA may subsequently undergo 7‐dehydroxylation to form LCA [28].

Species‐specific microbial transformations further contribute to distinct BA profiles. In rodents, taurine‐conjugated α/βMCAs are deconjugated by BSH to yield unconjugated α‐ and β‐MCA. α‐muricholic acid is mainly converted to murideoxycholic acid through 7‐dehydroxylation, whereas β‐MCA undergoes C‐6 epimerization to ω‐muricholic acid, followed by 7‐dehydroxylation to produce hyodeoxycholic acid (HDCA) [26]. This microbial transformation network shapes the characteristic rodent SBA profile and contributes to the regulation of host lipid metabolism and energy homeostasis. In ruminants, rumen microbiota extensively modifies PBAs before their entry into the small intestine, including 7β‐, 3β‐, 5α‐, and 6β‐epimerization as well as dehydroxylation. These rumen microbiota‐modified BAs may enter the blood circulation and contribute to the regulation of host amino acid and lipid metabolism [29].

### Regulation of T cell immunity by microbiota‐derived BAs

Recent evidence indicates that gut microbiota‐derived BA metabolites directly regulate T cell differentiation and function. After host‐synthesized PBAs enter the intestine, approximately 5%‐10% escape ileal reabsorption and reach the colon, where they are converted by microbial enzymes, including BSH, *bai* operon‐encoded enzymes, and HSDH, into a variety of secondary and modified BAs, such as DCA, LCA, 3‐oxoLCA, isoLCA, isoDCA, and isoalloLCA. These metabolites provide key molecular signals that modulate intestinal immunity [30]. In recent years, the relationship between BA metabolism and the balance between T helper 17 (Th17) cells and regulatory T (Treg) cells has received increasing attention. 3‐oxoLCA and isoLCA suppress Th17 differentiation by inhibiting RORγt activity. In human inflammatory bowel disease (IBD) cohorts, the levels of these BAs and the abundance of related HSDH genes are reduced and negatively associated with Th17‐related gene expression, suggesting that impaired microbial BA metabolism may contribute to the persistence of intestinal inflammation [31]. Consistently, recent reviews have emphasized that altered BA profiles in IBD are often accompanied by disruption of Th17/Treg homeostasis, further supporting the involvement of this metabolic axis in intestinal inflammation [32].

In parallel with the suppression of Th17 responses, several BA metabolites promote Treg generation. isoDCA acts mainly on dendritic cells, reducing antigen presentation and pro‐inflammatory signaling, thereby facilitating peripheral Foxp3^+^ Treg differentiation. Engineered microbial consortia capable of producing isoDCA also increase colonic RORγt^+^ Treg cells in vivo [33]. Another LCA derivative, isoalloLCA, promotes *Foxp3* expression through nuclear receptor NR4A1, and both isoalloLCA levels and its associated biosynthetic genes are decreased in patients with IBD, further indicating a direct link between microbial BA metabolism and Treg homeostasis [34]. In graft‐versus‐host disease, T cell‐driven inflammation reduces the abundance of BSH genes and microbiota‐derived BAs, thereby aggravating donor T cell‐mediated tissue injury. These findings suggest that disruption of BA metabolism itself may amplify T cell‐dependent inflammation [35].

However, the effects of BAs on T cells cannot be simply classified as anti‐inflammatory. In colorectal cancer, elevated DCA enhances plasma membrane Ca^2+^‐ATPase activity and suppresses Ca^2+^‐nuclear factor of activated T cells 2 signaling, thereby reducing the expression of granzyme B, IFN‐γ, and tumor necrosis factor‐α (TNF‐α) in CD8^+^ T cells, which weakens antitumor immunity and promotes tumor growth [36]. In studies of central nervous system autoimmunity, bacteria carrying enzymes involved in DCA/LCA production are reduced in patients with multiple sclerosis; decreased intestinal DCA is associated with increased Th17 cells, whereas DCA/LCA supplementation alleviates experimental autoimmune encephalomyelitis, indicating that the immunoregulatory effects of this pathway are not restricted to the intestine [37]. In addition, early‐life studies have shown that microbiota‐derived CBAs are associated with monocyte responses to lipopolysaccharide (LPS), the Th17/Treg balance, and the risk of islet autoimmunity, suggesting that BA metabolism may participate in shaping disease susceptibility during immune development [38]. Thus, the influence of gut microbiota‐derived BAs on T cells depends on the specific BA species, local concentration, target cell type, and disease context. Further longitudinal human studies and controlled microbiota‐intervention experiments are needed to define which BA metabolic pathways have stable and translatable therapeutic potential.

### Extrahepatic metabolism

BA precursors and signals act as critical inter‐organ messengers. While the liver is the primary site of synthesis, extrahepatic tissues such as the brain contribute significantly to the BA pool via the alternative biosynthetic pathway [21]. In the brain, the blood‐brain barrier (BBB) prevents direct cholesterol clearance. Instead, the brain‐specific enzyme cholesterol 24‐hydroxylase converts cholesterol into 24S‐hydroxycholesterol (24S‐HC) [39]. Being more polar than cholesterol, 24S‐HC efficiently crosses the BBB into systemic circulation. This process is facilitated by transporters such as ATP‐binding cassette transporters [40]. Upon reaching the liver, circulating 24S‐HC is 7α‐hydroxylated by 24‐hydroxycholesterol 7α‐hydroxylase. This modification directs it into the BA synthetic pathway, preferentially generating CDCA [41]. Furthermore, the enzymes oxysterol 7α‐hydroxylases are expressed in peripheral tissues such as the adrenal glands, participating in steroidogenic metabolism [42]. Collectively, these findings underscore the need to move beyond isolated organ studies and to analyze BA metabolism as a coordinated, multi‐organ network to fully understand its systemic physiological significance.

## 3 PHYSIOLOGICAL FUNCTIONS AND MOLECULAR MECHANISMS OF BA RECEPTORS

BA receptors are broadly categorized into nuclear receptors and membrane receptors. FXR and TGR5 are the most extensively characterized. Upon activation by BAs, these receptors regulate diverse physiological functions (Figure 2).

**Figure 2:**
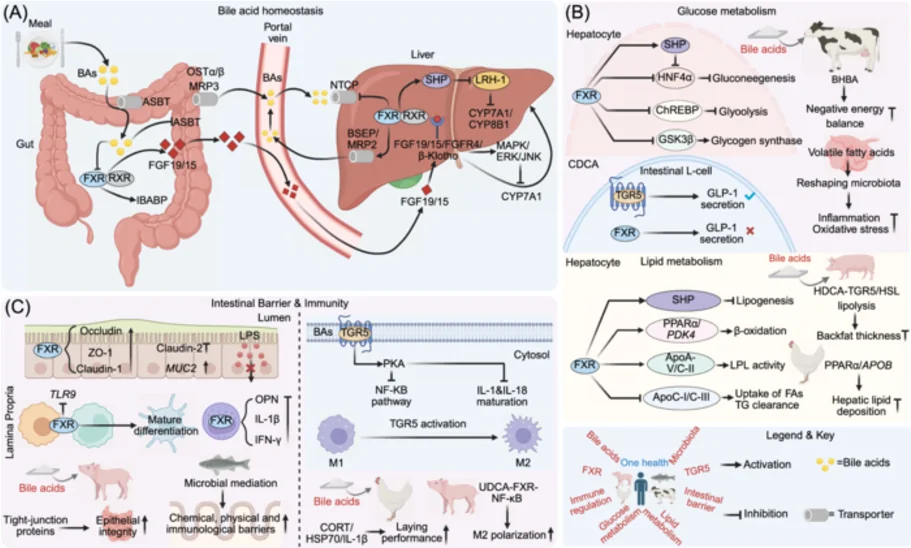
BA receptor‐mediated regulation of metabolic, barrier, and immune homeostasis. (A) After meals, bile acids (BAs) are reabsorbed in the intestine through apical sodium‐dependent bile acid transporter (ASBT), exported via organic solute transporter α/β (OSTα/β) and multidrug resistance‐associated protein 3 (MRP3), returned to the liver through portal circulation, and taken up by sodium taurocholate co‐transporting polypeptide (NTCP). Hepatic and intestinal farnesoid X receptor (FXR) activation induces small heterodimer partner (SHP) and fibroblast growth factor 19/15 (FGF19/15)‐fibroblast growth factor receptor 4 (FGFR4)/beta‐Klotho (β‐Klotho) signaling, thereby suppressing CYP7A1/CYP8B1 and maintaining BA homeostasis. (B) BA‐activated FXR and Takeda G protein‐coupled receptor 5 (TGR5) coordinate glucose and lipid metabolism by inhibiting gluconeogenesis and lipogenesis, promoting glycogen synthesis, glucagon‐like peptide‐1 (GLP‐1) secretion, fatty acid (FA) β‐oxidation, and triglyceride (TG) clearance. These mechanisms are linked to adaptation to negative energy balance in dairy cows, reduced fat deposition in pigs and poultry, and alleviated hepatic lipid accumulation. (C) In the intestine, FXR strengthens tight‐junction and mucin barriers, whereas FXR/TGR5 signaling suppresses nuclear factor kappa‐light‐chain‐enhancer of activated B cells (NF‐κB)/NOD‐like receptor family pyrin domain containing 3 (NLRP3)‐mediated inflammation and promotes M2 macrophage polarization, supporting barrier integrity, immune homeostasis, and production performance in pigs, poultry, and aquatic animals. ApoA‐V, apolipoprotein A‐V; APOB, apolipoprotein B; ApoC‐I, apolipoprotein C‐I; ApoC‐II, apolipoprotein C‐II; ApoC‐III, apolipoprotein C‐III; BHBA, β‐hydroxybutyrate; BSEP, bile salt export pump; CDCA, chenodeoxycholic acid; ChREBP, carbohydrate response element‐binding protein; CORT, corticosterone; ERK, extracellular signal‐regulated kinase; GSK3β, glycogen synthase kinase 3β; HDCA, hyodeoxycholic acid; HNF4α, hepatocyte nuclear factor 4α; HSL, hormone‐sensitive lipase; HSP70, heat shock protein 70; IBABP, ileal bile acid‐binding protein; IFN‐γ, interferon‐γ; IL‐1β, interleukin‐1β; IL‐18, interleukin‐18; JNK, c‐Jun N‐terminal kinase; LRH‐1, liver receptor homolog‐1; LPL, lipoprotein lipase; LPS, lipopolysaccharide; M1 macrophage, classically activated macrophage; M2 macrophage, alternatively activated macrophage; MAPK, mitogen‐activated protein kinase; MRP2, multidrug resistance‐associated protein 2; MUC2, mucin 2; OPN, osteopontin; PDK4, pyruvate dehydrogenase kinase 4; PKA, protein kinase A; PPARα, peroxisome proliferator‐activated receptor α; RXR, retinoid X receptor; TLR9, toll‐like receptor 9; UDCA, ursodeoxycholic acid; ZO‐1, zonula occludens‐1.

### Orchestration of BA homeostasis

Following a meal, intestinal BA concentrations rise. To prevent excessive *de novo* synthesis and potential hepatotoxicity, the liver orchestrates a robust negative feedback mechanism to maintain BA pool homeostasis [43] (Figure 2A). In hepatocytes, BA‐activated FXR forms a heterodimer with the retinoid X receptor. This complex binds to the promoter region of the small heterodimer partner (SHP) gene, inducing its transcription. SHP, a potent transcriptional repressor, subsequently binds to and inhibits liver receptor homolog‐1. This ultimately suppresses the transcription of key synthetic enzymes, *CYP7A1* and *CYP8B1* [44]. Within intestinal epithelial cells, BA‐activated FXR induces the expression and secretion of fibroblast growth factor 19 (FGF19, in humans) or FGF15 (in mice). Circulating FGF19/15 reaches the liver and binds to the fibroblast growth factor receptor 4 (FGFR4)/β‐Klotho co‐receptor complex on the hepatocyte surface [44]. This activation triggers intracellular kinase cascades, including the mitogen‐activated protein kinase/extracellular signal‐regulated kinase (MAPK/ERK) and c‐Jun N‐terminal kinase (JNK) pathways, which converge to repress CYP7A1 transcription, thereby reducing BA synthesis [45]. Furthermore, FXR coordinates BA transport to prevent hepatic accumulation. In the liver, FXR induces the expression of efflux transporters (BSEP and MRP2) and inhibits the uptake transporter (NTCP), collectively reducing intracellular BA concentrations and mitigating liver injury [46]. In the ileum, FXR downregulates the apical uptake transporter ASBT while upregulating the intracellular ileal bile acid‐binding protein. These coordinated actions precisely modulate ileal reabsorption efficiency, preserving the essential role of BAs in fat digestion while preventing toxicity from excessive accumulation [46]. Thus, FXR is a master regulator of the enterohepatic circulation, ensuring BA pool stability.

### Regulation of glucose and lipid metabolism

The BA‐FXR and TGR5 signaling pathways in the liver and intestine are pivotal regulators of glucose metabolism. They govern gluconeogenesis, glycogen synthesis, and insulin sensitivity [47] (Figure 2B). Upon activation by BAs in the liver, FXR induces the expression of SHP. SHP subsequently interacts with and inhibits transcription factors, such as hepatocyte nuclear factor 4α, thereby disrupting their recruitment of the coactivator peroxisome proliferator‐activated receptor γ coactivator 1α [48]. This interference suppresses the expression of key gluconeogenic enzymes, including phosphoenolpyruvate carboxykinase and glucose‐6‐phosphatase, thereby reducing hepatic gluconeogenesis and lowering blood glucose levels [48]. Additionally, activated FXR inhibits the transcriptional activity of carbohydrate response element‐binding protein, suppressing glucose‐induced glycolytic genes such as liver pyruvate kinase [49]. Furthermore, FXR promotes glycogen synthesis, thereby increasing hepatic glycogen content. This is mediated by the phosphorylation and subsequent inactivation of glycogen synthase kinase 3β, which relieves inhibition of glycogen synthesis [50].

Intestinal BA‐FXR signaling induces the secretion of FGF19/FGF15, which binds to the hepatic FGFR4/β‐Klotho complex. This signaling axis exerts insulin‐sensitizing and glucose‐lowering effects, inhibiting gluconeogenesis and stimulating glycogen synthesis and energy expenditure [45]. Consistently, *FGF15* knockout mice exhibit hyperglycemia and impaired hepatic glycogen storage, while FGF19 administration rescues these metabolic phenotypes, underscoring the beneficial role of the FGF19/15 axis in glucose homeostasis [51]. In the intestine, CDCA activates TGR5 on the membrane of L cells, triggering the conversion of ATP to cyclic adenosine monophosphate (cAMP) and subsequent activation of protein kinase A (PKA) [52, 53]. PKA‐mediated phosphorylation promotes the maturation and secretion of glucagon‐like peptide‐1 (GLP‐1) and triggers the opening of voltage‐dependent calcium channels. The resulting influx of Ca^2+^ stimulates the rapid exocytosis of GLP‐1, improving glucose homeostasis [8]. Conversely, intestinal FXR activation has been reported to inhibit GLP‐1 secretion by suppressing proglucagon transcription [54]. While TGR5 activation elicits a rapid postprandial response, FXR induces delayed transcriptional repression [54]. This creates a temporal interplay where the acute positive effect of TGR5 on GLP‐1 secretion may be subsequently counterbalanced by FXR‐mediated inhibition.

BAs are intricately linked to lipid metabolism. In the liver, FXR activation induces SHP expression, which subsequently interacts with and inhibits the activity of liver X receptor α and hepatocyte nuclear factor 4α [48]. This transcriptional repression downregulates the sterol regulatory element‐binding protein 1c (SREBP‐1c) and its downstream target enzymes, fatty acid (FA) synthase and acetyl‐CoA carboxylase, leading to decreased *de novo* FA and triglyceride (TG) synthesis [48]. Concurrently, FXR upregulates the expression of peroxisome proliferator‐activated receptor α (PPARα) and pyruvate dehydrogenase kinase‐4. This promotes FA β‐oxidation in mitochondria and peroxisomes, effectively reducing hepatic and systemic lipid levels [55]. Furthermore, FXR activation enhances plasma lipoprotein lipase activity by inducing the expression of its activators, apolipoprotein (Apo) A‐V and ApoC‐II, while suppressing its inhibitors, ApoC‐I and ApoC‐III. These coordinated changes promote FA uptake by tissues and the clearance of TG [56]. Within the gut‐liver axis, the FGF19/15 signaling pathway (mediated by the FGFR4/β‐Klotho complex) further suppresses SREBP‐1c activity. This not only reduces hepatic de novo lipogenesis but also represses BA synthesis from cholesterol, thereby exerting a comprehensive regulatory effect on lipid metabolism [57].

### Role in intestinal barrier integrity and immune homeostasis

Regarding the physical barrier, FXR activation promotes the transcription and expression of tight junction proteins such as Occludin, Zonula Occludens‐1, and Claudin‐1, while inhibiting the expression of Claudin‐2 (Figure 2C). These changes strengthen the connections between intestinal epithelial cells and reduce intestinal permeability [58, 59]. Consistently, FXR activation (or potent agonists) has been shown to alleviate intestinal barrier dysfunction and reduce inflammatory damage caused by LPS, thereby maintaining tight junction integrity [60]. Regarding the chemical barrier, BAs and FXR signaling are critical for mucin production. FXR activation promotes the expression of Mucin 2 (MUC2), whereas FXR knockout or inhibition leads to decreased MUC2 levels in mouse intestinal organoids [61]. For instance, CDCA activates FXR in gastric epithelial cells in a dose‐dependent manner, upregulating MUC2 protein expression [62]. Furthermore, tauroursodeoxycholic acid alleviates endoplasmic reticulum stress, stimulates goblet cell mucus secretion, and increases mucus layer thickness, thereby enhancing the chemical barrier [63]. In the context of inflammation, reduced FXR signaling impairs transporter function, leading to hepatic BA accumulation and persistent inflammation [26]. FXR and TGR5 are widely expressed in immune cells, including monocytes, macrophages, dendritic cells, natural killer cells, and T cells [64, 65, 66]. FXR activation in monocytes and macrophages inhibits Toll‐like receptor 9 (TLR9)‐dependent pro‐inflammatory responses and regulates the differentiation of CD14^+^ monocytes into mature dendritic cells [66, 67]. In natural killer T cells, FXR activation suppresses the production of osteopontin, interleukin‐1β (IL‐1β), and interferon‐γ [9]. Mechanistically, FXR exerts anti‐inflammatory effects by antagonizing nuclear factor kappa‐B (NF‐κB) activity through trans‐repression. FXR stabilizes nuclear corepressor complexes at the promoters of pro‐inflammatory genes (e.g., tumor necrosis factor‐α (TNF‐α) and IL‐1β), thereby preventing NF‐κB binding and transcriptional activation [68, 69].

TGR5 exerts anti‐inflammatory effects primarily through the cAMP‐PKA pathway, which subsequently activates cAMP response element‐binding protein. In monocytes and macrophages, TGR5 activation stimulates adenylate cyclase, thereby increasing intracellular cAMP levels. cAMP activates PKA, which phosphorylates downstream targets to inhibit the NF‐κB signaling pathway, potentially involving signal transducer and activator of transcription 1‐dependent mechanisms, and consequently reduces the secretion of cytokines such as IL‐6, IL‐8, and TNF‐α [70]. Furthermore, the TGR5‐cAMP‐PKA axis blocks the assembly and activation of the NOD‐like receptor family pyrin domain‐containing 3 (NLRP3) inflammasome by promoting NLRP3 ubiquitination, thereby preventing the maturation and release of IL‐1β and IL‐18 [71]. Additionally, TGR5 promotes macrophage polarization from the pro‐inflammatory M1 to the anti‐inflammatory M2 phenotype [72]. Conversely, TGR5 deficiency exacerbates liver injury and promotes M1 polarization by facilitating NLRP3 inflammasome activation and caspase‐1 cleavage [73].

## 4 MULTIPLE FACTORS AFFECTING BA METABOLISM

### Population diversity

BA metabolism is markedly altered in diabetes and MASLD, and these changes influence host glucose and lipid metabolism, inflammatory responses, and intestinal barrier function through interactions with the gut microbiota (Figure 3A). Studies have shown that insulin resistance and type 2 diabetes (T2DM) are commonly accompanied by increased BA synthesis, altered BA composition, and impaired enterohepatic circulation. In this context, disturbed glucose and insulin signaling may promote CYP8B1‐mediated production of 12α‐hydroxylated BAs, which are closely associated with hepatic insulin resistance, suggesting that BAs may contribute to the progression of metabolic dysfunction rather than merely reflecting it [74]. In MASLD, dysregulated BA signaling is mainly characterized by impaired FXR‐FGF19 feedback, increased CYP7A1 activity, upregulation of NTCP, and downregulation of BSEP, thereby increasing hepatocellular BA burden and interacting with lipotoxicity, intestinal barrier injury, inflammation, and fibrosis [75]. In addition, fecal BSH activity increases with fibrosis severity in patients with MASLD, whereas total fecal BA levels decrease. Unconjugated BAs can disrupt the intestinal epithelial barrier, and BSH inhibitors are able to reduce BA deconjugation activity in patient‐derived microbial communities, indicating that microbial BSH may represent an important therapeutic target along the gut‐liver axis in MASLD [76].

**Figure 3:**
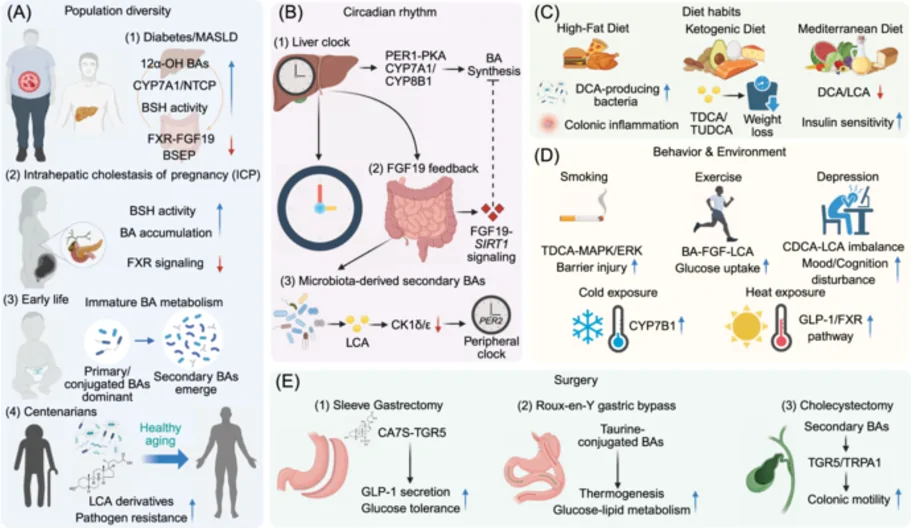
Multiple factors dynamically shape bile acid (BA) metabolism. BA metabolism is influenced by population diversity, circadian rhythm, diet, behavior, environmental exposure, and surgical intervention. (A) Different physiological and pathological states, including diabetes/metabolic dysfunction‐associated steatotic liver disease (MASLD), intrahepatic cholestasis of pregnancy, early life and healthy aging, are associated with distinct BA profiles. (B) Circadian rhythm and (C) dietary patterns further modulate BA composition, microbial transformation, and host metabolic responses. (D) Smoking, exercise, mental status, and temperature exposure reshape BA‐related signaling in a context‐dependent manner. (E) Surgical procedures, including sleeve gastrectomy, Roux‐en‐Y gastric bypass, and cholecystectomy, remodel postoperative BA pools and downstream physiological outcomes. Blue arrows represent an increase, and red arrows represent a decrease. 12α‐OH BA, 12α‐hydroxylated bile acid; BSEP, bile salt export pump; BSH, bile salt hydrolase; CA7S, cholic acid‐7‐sulfate; CDCA, chenodeoxycholic acid; CK1δ/ε, casein kinase 1δ/ε; CYP7A1, cholesterol 7α‐hydroxylase; CYP7B1, oxysterol 7α‐hydroxylase; CYP8B1, sterol 12α‐hydroxylase; DCA, deoxycholic acid; ERK, extracellular signal‐regulated kinase; FGF, fibroblast growth factor; FGF19, fibroblast growth factor 19; FXR, farnesoid X receptor; GLP‐1, glucagon‐like peptide‐1; LCA, lithocholic acid; MAPK, mitogen‐activated protein kinase; NTCP, sodium taurocholate co‐transporting polypeptide; PER1, period circadian regulator 1; PER2, period circadian regulator 2; PKA, protein kinase A; SIRT1, sirtuin 1; TDCA, taurodeoxycholic acid; TGR5, Takeda G protein‐coupled receptor 5; TRPA1, transient receptor potential ankyrin 1; TUDCA, tauroursodeoxycholic acid.

Pregnancy‐associated cholestasis further highlights the pathological relevance of the microbiota‐BA‐FXR pathway. Patients with intrahepatic cholestasis of pregnancy show enrichment of *Bacteroides fragilis*, whose BSH activity removes taurine or glycine from conjugated BAs and suppresses FXR signaling, leading to increased BA synthesis, impaired hepatobiliary excretion, and placental and fetal abnormalities [77]. Large cohort studies have also demonstrated that higher maternal serum total BA levels are associated with a higher risk of preterm birth; activation of FXR and restoration of BA homeostasis reduce preterm birth and improve neonatal survival, supporting a direct contribution of BA dysregulation to adverse pregnancy outcomes [78]. In early life, BAs are closely linked to gut microbiota maturation. The gradual establishment of hepatic BA synthesis and transport in newborns occurs in parallel with intestinal microbial succession; in mice, supplementation with taurocholic acid (TCA) or β‐tauromuricholic acid accelerates neonatal gut microbiota maturation [79]. Studies in human infants have shown that fecal BA profiles are initially dominated by conjugated or primary BAs, followed by the appearance of transformed products such as UDCA. With the introduction of solid foods and the expansion of intestinal anaerobes, microbially derived secondary BAs, including DCA and LCA, progressively increase [80]. Notably, in children at risk of type 1 diabetes, alterations in microbial secondary BA metabolic pathways precede the appearance of islet autoantibodies, suggesting that early‐life BA metabolic changes may be associated with susceptibility to immune‐metabolic disease [81]. In extremely premature infants with brain injury, changes in plasma and fecal BAs, amino acid‐conjugated BAs, and neuroactive metabolites are already detectable within the first days of life, indicating that BAs may participate in early interactions among the gut microbiota, immune system, and neurodevelopment [82].

In contrast to disease‐associated BA disturbances, studies of centenarians suggest that certain BA changes may be linked to healthy aging. Their gut microbiota is enriched in taxa such as *Clostridium scindens* and Odoribacteraceae that are capable of producing LCA derivatives, including isoLCA, 3‐oxoLCA, alloLCA, and isoalloLCA. Among these, isoalloLCA is generated through the actions of 5α‐reductase and 3β‐hydroxysteroid dehydrogenase and exhibits inhibitory activity against Gram‐positive pathogens such as *Clostridioides difficile* (*C. difficile*) [83]. Moreover, the centenarian gut virome is enriched in auxiliary metabolic genes related to sulfur metabolism, and serum metabolomic analyses show that higher levels of BAs such as CDCA and LCA are associated with reduced mortality risk [84, 85]. These findings indicate that the biological effects of BAs cannot be explained simply by total BA abundance; rather, they depend on specific BA species, microbial metabolic capacity, host physiological context, and the maintenance of intestinal mucosal homeostasis.

Sex is an important determinant of BA metabolism, yet the direction of its regulatory effects differs markedly between rodents and humans. In humans, BA synthesis varies by more than nine‐fold among healthy individuals; men exhibit approximately 29%–30% higher BA synthesis than women, and this variation is positively associated with serum TG but inversely associated with circulating FGF19 levels [86]. These findings suggest that the intestinal FXR–FGF19–hepatic CYP7A1 feedback axis represents a key node linking sex‐dependent BA regulation with lipid homeostasis. However, this sexual dimorphism is clearly species dependent. In mice, females instead display a larger BA pool and a composition relatively enriched in CDCA‐derived species [87]. Such interspecies divergence highlights the need for caution when extrapolating findings from animal models to humans. Moreover, estrogens may upregulate CYP7B1 through estrogen receptor α while suppressing CYP8B1‐related signaling, whereas *CYP3A4* expression and activity are generally higher in women; this difference becomes attenuated after menopause as estrogen levels decline [87]. Beyond host endocrine regulation, the gut microbiota further imposes sex specificity on BA metabolism. A recent portal BA profiling study showed that oxidized BAs in the portal circulation of male mice were approximately three‐fold higher than those in females, whereas conjugated PBAs were approximately 1.8‐fold more abundant in females than in males [88]. These differences disappeared in germ‐free or defined‐community mice, indicating that microbiota‐mediated deconjugation, oxidation, and SBA transformation are major contributors to sex‐specific enterohepatic cycling [88].

Pregnancy further amplifies the plasticity of BA metabolism. In pregnant rats, hepatic BA homeostasis can be maintained through FXR–SHP feedback, which may help limit excessive fetal exposure to BAs [89]. However, when this compensatory mechanism is disrupted by genetic or endocrine factors, intrahepatic cholestasis of pregnancy may occur [89, 90]. Lactation presents a distinct metabolic state. To meet the high energetic demands of milk production, lactating rats exhibit increased hepatic BA accumulation, accompanied by upregulation of *CYP7A1*, *CYP8B1*, *CYP27A1*, *CYP7B1* and the BA transporters BSEP, MRP3, and MRP4 [89]. These changes indicate that the liver enhances BA synthesis while simultaneously strengthening biliary and basolateral efflux capacity. Recent studies have further shown that BAs in breast milk are not derived solely from hepatic–intestinal circulation, but can also be transported directly from the maternal intestine to the mammary gland through an “entero‐mammary axis” [90, 91]. Notably, breast‐milk BAs exert dual regulatory effects on the neonate. On the one hand, milk enriched in taurine‐conjugated PBAs can activate neonatal TGR5 signaling and promote insulin‐like growth factor‐1 production, thereby supporting postnatal growth [91]. On the other hand, these CBAs may also create a favorable environment for the replication of enteric pathogens such as norovirus, increasing neonatal susceptibility to viral diarrhea [90]. Therefore, breast‐milk BAs should be viewed not only as endocrine signals that promote growth, but also as critical regulators shaping neonatal gut microbial ecology and anti‐infective defense.

### Circadian rhythm

Circadian rhythm is closely involved in the daily regulation of BA metabolism. Rather than acting through a single pathway, it affects BA synthesis, secretion, enterohepatic circulation, and microbial transformation through the combined influence of light‐dark cycles, sleep‐wake behavior, feeding time, and diet composition [92] (Figure 3B). These rhythmic cues shape the intestinal microbial community and its metabolic activity, thereby linking host timekeeping with microbial BA metabolism [93]. In healthy men maintained under controlled laboratory conditions, circulating BAs showed clear 24‐h fluctuations. Conjugated BAs, which mainly reflect hepatic secretion, generally appeared earlier, whereas unconjugated BAs, which are closely related to microbial deconjugation, peaked several hours later [94]. This phase difference suggests that host‐derived BA output and microbial BA processing are temporally separated. Sleep deprivation did not completely abolish BA rhythmicity, but it disrupted the temporal association between BAs and plasma lipids [94]. By contrast, when environmental timing cues were kept constant, BA rhythmicity was largely lost, indicating that feeding‐fasting and sleep‐wake cycles are major drivers of circulating BA oscillations.

The liver clock provides an important mechanistic basis for this phenomenon. Period circadian regulator 1 (*PER1*) has been shown to interact directly with CYP7A1 and CYP8B1, two key enzymes in BA synthesis, and to regulate their activity and stability through a *PER1*‐PKA‐dependent phosphorylation pathway [95]. In *PER1*‐deficient mice, the BA pool was reduced, the day‐night difference in intestinal fat absorption was weakened, and susceptibility to high‐fat diet (HFD) ‐induced obesity was decreased; supplementation with BAs restored lipid absorption, supporting a functional link between circadian BA synthesis and time‐dependent fat handling [95]. The gut‐liver endocrine feedback also participates in this temporal regulation. Postprandial BA flux induces intestinal FGF15/19, which feeds back to inhibit hepatic BA synthesis. In night‐shift workers, the rhythms of plasma FGF19 and total BAs were disturbed, accompanied by altered NAD^+^‐SIRT1 signaling, suggesting that chronic circadian misalignment can reshape BA and energy metabolism through the FGF19‐SIRT1 axis [96].

Gut microbiota further modifies this rhythmic system by converting primary BAs into secondary BAs [97]. LCA, a microbiota‐derived secondary BA, lengthened the human PER2 transcriptional period in human colonic cells, inhibited CK1δ/ε, affected CK1δ/ε‐protein phosphatase 1 signaling, and stabilized CRY2, indicating that microbial BA metabolites can directly influence peripheral clock machinery [98]. This relationship is bidirectional. In chronic liver disease, elevated systemic BAs were associated with circadian rhythm sleep disorders; mechanistically, BAs entered the suprachiasmatic nucleus and activated the TGR5‐ERK‐CK1ε‐PER2 pathway, thereby disturbing central clock function [99]. Collectively, current evidence suggests that BA rhythmicity is shaped by behavioral timing, hepatic clock genes, FGF15/19 feedback, SIRT1‐dependent energy sensing, and microbiota‐derived secondary BAs. However, BA composition and microbial BA transformation differ substantially between rodents and humans; more time‐resolved human studies are still needed.

### Dietary habits

Dietary composition serves as a key driver in shaping the gut microbial community and its metabolic functions. BAs, as core products of host‐microbiota co‐metabolism, have emerged as an important hub linking diet, the gut microbiome, and host metabolic health. Different dietary patterns profoundly influence the pool size, composition, and signaling activity of BAs by modulating the abundance and function of specific microbial taxa (Figure 3C). For example, long‐term HFD feeding induces overgrowth of Gram‐positive bacteria, particularly those capable of producing deoxycholic acid (DCA; e.g., *Clostridium* spp.), leading to accumulation of this SBA in the enterohepatic circulation and feces. DCA subsequently skews macrophage polarization toward a pro‐inflammatory M1 phenotype through the M2 muscarinic acetylcholine receptor and Toll‐like receptor 2 pathways, driving colonic inflammation and tissue injury [100]. In contrast, a ketogenic diet (KD) reduces the abundance of the BSH‐encoding bacterium *Lactobacillus murinus ASF361*, thereby elevating circulating levels of taurine‐CBAs such as taurodeoxycholic acid (TDCA) and tauroursodeoxycholic acid (TUDCA). These CBAs inhibit intestinal carbonic anhydrase 1 expression and reduce caloric absorption, contributing to weight loss. However, KD also decreases overall BSH gene richness and short‐chain fatty acid (SCFA) levels, thereby disrupting gut microbial diversity [101]. Moreover, if KD promotes colonization by *Clostridium perfringens*, this bacterium accelerates the conversion of DCA to 12‐ketolithocholic acid, which exacerbates hepatic ketogenesis and steatosis via the BA‐FXR‐PPARα axis and upregulation of DGAT2 [102]. Thus, the same dietary regimen can exert both beneficial and deleterious effects on the microbiota‐BA network in a strain‐ and pathway‐specific manner, and disentangling these opposing effects represents a central challenge for future precision nutrition research. Interestingly, weight regain after caloric restriction is closely associated with a persistent decrease in *Parabacteroides distasonis* and depletion of non‐12α‐hydroxylated BAs such as UDCA. Supplementation with *Parabacteroides distasonis* or UDCA restores thermogenesis and counteracts weight regain [103]. Furthermore, even a low‐fat refined diet (high in sugar and low in fiber) triggers BA dysmetabolism that extends to the central nervous system, leading to reduced levels of CA and TCA in the brain, which in turn relieves inhibition of the hippocampal transcription factor NF‐κB1 and activates neuroinflammatory signaling [104]. These findings expand our understanding of the diet‐gut‐brain axis; however, the precise mechanisms by which BAs cross the BBB and their potential for local synthesis in the brain remain to be elucidated. By contrast, a plant‐rich Mediterranean diet exhibits broad protective effects. In overweight and obese individuals, it enriches fiber‐degrading beneficial bacteria such as *Faecalibacterium prausnitzii*, increases SCFA production, reduces levels of DCA and LCA, and ameliorates low‐grade inflammation and improves insulin sensitivity without altering total energy intake. In elderly populations, the Mediterranean diet also promotes healthy aging [105, 106]. Nevertheless, current evidence remains largely correlative; future studies integrating humanized gnotobiotic models and randomized controlled trials are needed to establish causality and to validate and extend these findings.

### Behavior, emotions, and environmental exposure

Smoking, exercise, mental status, and thermal‐humidity environments can markedly influence the gut microbiota and BA metabolism, with different exposures inducing distinct alterations in BA profiles, microbial functions, and host physiological responses. Cigarette smoke exposure disrupts hepatic cholesterol and BA homeostasis in mice, as evidenced by hepatic cholesterol accumulation, reduced *CYP8B1* expression, a decreased CA/CDCA ratio, and concomitant gut microbial dysbiosis [107] (Figure 3D). In a colorectal cancer model, smoke exposure further enriched *Eggerthella lenta*, increased TDCA levels, activated MAPK/ERK signaling, and impaired intestinal barrier integrity [108]. These findings suggest that the effects of smoking on the distal gut may not result solely from direct toxicity, but may also be amplified through a microbiota‐BA‐epithelial signaling axis that promotes inflammation and tumorigenesis.

In contrast, exercise generally appears to modulate the BA axis in an adaptive manner. Human studies have shown that total BA levels often decrease after acute exercise [109]. However, endurance and resistance exercise exert mode‐specific effects on the BA‐FGF axis. Endurance exercise increases FGF21, whereas resistance exercise decreases FGF19 and total BA levels, and both exercise modes can increase LCA [110]. Mouse studies further demonstrate that exercise‐acclimated microbiota enhances BA deconjugation, activates AMPK signaling in skeletal muscle, and improves glucose uptake [111]. In osteopenic models, UDCA, taurine, and apelin signaling are involved in exercise‐associated bone‐protective effects [112]. Together, these findings indicate that the BA axis may serve as an important mediator through which exercise‐induced gut microbial changes are transmitted to skeletal muscle and bone.

In patients with major depressive disorder, BA dysmetabolism is associated with cognitive impairment, anxiety severity, and treatment response, characterized mainly by reduced CDCA, increased LCA‐related secondary BAs, and alterations in microbial taxa such as Lachnospiraceae, Ruminococcaceae, and *Escherichia coli* [113, 114]. TGR5 signaling can regulate the lateral hypothalamus‐hippocampal CA3‐dorsolateral septum circuit, suggesting that BA signaling may directly participate in the regulation of stress‐related and emotional behaviors [115]. The effects of environmental temperature and humidity are more context dependent: humid heat exposure can alter LCA levels and GLP‐1/FXR signaling and induce anxiety‐like or metabolic phenotypes [116, 117]. Conversely, cold exposure promotes cholesterol‐to‐BA conversion through a CYP7B1‐dependent pathway and supports adaptive thermogenesis [118]. However, under chronic cold exposure in the context of arthritis, reduced taurohyodeoxycholic acid may exacerbate inflammation [119]. Thus, the effects of the BA axis are highly context dependent and are shaped by exposure type, exposure duration, and host status. Future studies integrating longitudinal human cohorts, controlled interventions, and mechanistic validation are needed to define the causal roles of key microbial taxa and BA species.

### Surgery

Bariatric surgery and cholecystectomy can both profoundly alter the gut microbiota and host metabolic responses by reshaping the enterohepatic circulation of BAs (Figure 3E). Vertical sleeve gastrectomy does not simply depend on hepatic or intestinal FXR activation; instead, it reduces small‐intestinal BA levels and intestinal lipid absorption. Supplementation with TCA partially attenuates the metabolic benefits of vertical sleeve gastrectomy, suggesting that reduced luminal BA availability itself contributes to postoperative improvements in body weight and glucose and lipid metabolism [120]. Notably, specific BA species are selectively altered after sleeve gastrectomy. Cholic acid‐7‐sulfate (CA7S) is increased in the intestine of mice and in human feces after surgery, where it functions as a gut‐restricted TGR5 agonist that stimulates GLP‐1 secretion and improves glucose tolerance [121]. In parallel, elevated small‐intestinal TDCA suppresses the bacterial *bai* operon and reduces LCA production, whereas LCA has been shown to aggravate glucose intolerance and adiposity. These findings indicate that the effects of BAs after bariatric surgery are highly dependent on specific molecular species and anatomical location [122]. In Roux‐en‐Y gastric bypass models, functional remodeling of the postoperative microbiota promotes the accumulation of taurine‐conjugated BAs, which reactivate thermogenic brown adipose tissue and improve glucose and lipid metabolism through intestinal FXR and systemic TGR5 signaling [123]. In addition, sleeve gastrectomy enriches gut commensal Clostridia and increases conjugated secondary BAs, including glycodeoxycholic acid and TDCA, which are associated with body‐weight reduction. Fecal microbiota transplantation and glycodeoxycholic acid supplementation both promote lipolysis and FA oxidation, partly through TGR5‐dependent mechanisms [124].

By contrast, in post‐cholecystectomy diarrhea, patient‐derived microbiota accelerates intestinal transit and increases fecal water content in mice. Secondary BAs, including DCA, HDCA and LCA, stimulate colonic 5‐hydroxytryptamine production through TGR5/transient receptor potential ankyrin 1 signaling, thereby enhancing colonic motility [125]. However, in dextran sulfate sodium‐induced colitis models, cholecystectomy‐induced accumulation of LCA, DCA, and HDCA suppresses monocyte/macrophage recruitment and promotes mucosal repair [126]. Conversely, in the context of colorectal tumorigenesis, cholecystectomy‐associated dysbiosis, characterized by reduced *Bifidobacterium breve* and increased *Ruminococcus gnavus*, is accompanied by elevated glycoursodeoxycholic acid and TUDCA, leading to FXR inhibition, disruption of the FXR‐β‐catenin interaction, and enhanced tumor development [127]. Thus, postoperative BA metabolism cannot be interpreted simply as an increase or decrease in total BA abundance. Instead, it should be understood in terms of the integrated effects of BA molecular species, intestinal compartmentalization, microbial biotransformation capacity, and host receptor pathways, including FXR, TGR5, and transient receptor potential ankyrin 1.

## 5 APPLICATIONS OF BAS IN HUMAN HEALTH

Despite meaningful clinical progress achieved with novel pharmacological agents such as resmetirom and semaglutide, the increasing burden of human liver and metabolic diseases underscores the pressing need for innovative therapeutic strategies [3, 128]. BAs show therapeutic potential in hepatobiliary diseases, metabolic disorders, neurodegenerative and retinal degenerative diseases, and intestinal infections. Their diverse clinical applications highlight the value of targeting BA signaling to improve health outcomes across multiple domains.

### Primary biliary cholangitis

BA‑based therapies have formed the backbone of drug development for primary biliary cholangitis (PBC) [129, 130]. Recent studies further indicate that BAs are not merely downstream products of cholestasis but serve as key mediators linking bile duct injury, intrahepatic inflammation, fibrosis progression, and pruritus [129, 130, 131, 132]. UDCA remains the foundation of all treatment; by increasing the proportion of hydrophilic BAs, improving bile secretion, and mitigating the toxicity of hydrophobic BAs to cholangiocytes, UDCA has profoundly changed the natural history of PBC [129, 130]. Nevertheless, residual risk after UDCA therapy cannot be fully explained by traditional criteria of “adequate response.” Recent studies on “deep response” have shown that even among patients who meet the conventional Paris‑2 criteria, normalization of alkaline phosphatase (ALP) is associated with better complication‑free survival, particularly in younger patients or those with existing fibrosis risk [133]. Thus, treatment goals for PBC are shifting from simply reducing cholestatic markers to more thoroughly alleviating BA‑driven persistent injury.

The FXR‐FGF19 axis has provided important mechanistic insight into the regulation of BA synthesis [129, 130]. Obeticholic acid (OCA), an FXR agonist, induces intestinal FGF19 and suppresses CYP7A1, thereby reducing de novo BA synthesis and improving ALP levels in patients with an incomplete response to UDCA [129, 130]. However, its clinical use needs to be individualized based on pruritus burden and disease stage; both the POISE trial and real‑world data indicate that pruritus is a common adverse effect of OCA, and special safety concerns apply to patients with advanced cirrhosis or portal hypertension [130, 134]. NGM282, a non‑tumorigenic FGF19 analog, validates this pathway from a different angle: in a short‑term PBC study, it reduced ALP and aminotransferases [135]; in a primary sclerosing cholangitis study, although it did not significantly lower ALP, it decreased C4, BAs, and fibrosis‑related biomarkers [136]. These findings suggest that ALP, while being the most practical surrogate endpoint, does not fully capture changes in BA synthesis, intrahepatic inflammation, or fibrotic activity.

PPAR agonists are characterized by their ability not only to influence BA production but also to improve hepatocellular transport, detoxification, and inflammatory/metabolic status [130, 137, 138]. Fenofibrate, through PPARα activation, up‑regulates BA glucuronidation and the corresponding efflux processes, resulting in a decrease in total BAs and an increase in glucuronidated BAs, suggesting that “BA detoxification capacity” may contribute to its therapeutic efficacy [139]. Elafibranor, a dual PPARα/δ agonist, reduced ALP, gamma‐glutamyl transferase, and inflammatory markers in patients with an inadequate UDCA response [137]; patient‑reported outcomes from the ELATIVE trial also indicated possible improvement in pruritus and quality of life, though this needs to be interpreted cautiously in light of placebo effects on symptom endpoints [140]. Data on seladelpar further link BA metabolism to symptom mechanisms: a 52‑week study showed dose‑dependent improvement in cholestatic biochemical markers [141], and the IL‑31 substudy revealed that the decrease in IL‑31 correlated with reductions in BAs and pruritus, suggesting that pruritus in PBC may involve the interplay of BAs, immune cytokines, and sensory neural pathways [132]. Saroglitazar also demonstrated rapid ALP reduction, but some patients developed elevated aminotransferases, indicating that broader PPAR modulation still requires careful dose selection to balance efficacy and hepatic safety [138].

In contrast to these disease‑modifying strategies, linerixibat is primarily aimed at symptom burden. By inhibiting the ileal bile acid transporter (IBAT) and reducing BA reabsorption, linerixibat improved moderate‑to‑severe cholestatic pruritus and sleep interference in the full publication and the meeting abstract of the phase 3 GLISTEN trial [131, 142]. However, its effect on ALP and bilirubin is limited, and diarrhea is common; thus it is more appropriate as a pruritus‑control agent rather than a substitute for anti‑cholestatic therapy [131, 142]. In summary, UDCA reduces baseline BA toxicity, the FXR/FGF19 pathway suppresses de novo BA synthesis, PPAR agonists enhance handling and detoxification, and IBAT inhibitors relieve pruritus. A more rational future direction is to select combinations based on residual cholestasis, fibrosis stage, pruritus burden, and the safety margin in cirrhosis, rather than funneling all patients with an inadequate UDCA response into a single second‑line treatment pathway.

### Metabolic dysfunction‐associated steatotic liver disease

BA dysregulation in MASLD is not merely a secondary consequence of lipid accumulation, but rather a key metabolic signal linking hepatocellular lipotoxicity, microcholestasis, intestinal barrier dysfunction, inflammatory amplification, and fibrotic progression. BA receptors, particularly the FXR‐FGF19 axis, regulate BA synthesis, lipid and glucose metabolism, and gut‐liver barrier integrity, making this pathway one of the earliest and most systematically validated BA‐related therapeutic nodes in metabolic dysfunction‐associated steatohepatitis (MASH) [75].

OCA established the conceptual foundation for FXR agonism in MASH. Clinical studies have shown that OCA has potential antifibrotic activity; however, it has not consistently achieved MASH resolution, and its further development has been constrained by pruritus, increased low‐density lipoprotein cholesterol (LDL‐C), gallstone‐related events, and liver safety concerns [143]. These findings suggest that FXR agonism may represent an intervention biased toward antifibrotic activity rather than a monotherapy capable of remodeling the full pathological spectrum of MASH. Notably, the mechanisms of OCA are not limited to canonical FXR signaling. Preclinical studies indicate that OCA can suppress NLRP3 inflammasome activation in macrophages [144], whereas gut microbiome studies suggest that its therapeutic efficacy is influenced by microbiota‐mediated remodeling of the BA pool [145]. In addition, self‐assembled nanocrystals composed of OCA and atorvastatin suggest that liver‐targeted delivery, combined with simultaneous mitigation of lipid‐related adverse effects, may represent a strategy for redesigning FXR‐based therapies, although its translational value remains to be validated in humans [146].

Non‐BA FXR agonists have been developed to improve the potency‐tolerability trade‐off observed with OCA. Cilofexor reduces magnetic resonance imaging‐proton density fat fraction (MRI‐PDFF), gamma‐glutamyl transferase, C4, and primary BAs, but has not shown clear improvement in elastography‐ or fibrosis‐related endpoints [147]. Tropifexor lowers alanine aminotransferase (ALT) and hepatic fat content; however, dose‐dependent pruritus and lipid profile alterations remain prominent, underscoring the narrow therapeutic window between FXR activation and tolerability [148]. MET409 provides evidence that structural optimization may improve this balance: it significantly reduced hepatic fat content within 12 weeks, and the 50 mg dose showed a relatively favorable pruritus and LDL‐C profile [149]. These data suggest that FXR agonists should not be viewed as a uniformly failed drug class, but rather as agents whose therapeutic window may be redefined through tissue selectivity, pharmacokinetic optimization, and dose design. The combination of the FXR agonist TERN‐101 with the thyroid hormone receptor β agonist TERN‐501 maintained or modestly enhanced improvements in MRI‐PDFF and corrected T1 without introducing new safety signals, further supporting the concept that FXR modulation may be more suitable as a metabolic component of combination therapy than as a stand‐alone approach to address all pathological dimensions of MASH [150].

FGF19 analogs bypass upstream FXR activation and directly test whether suppression of de novo BA synthesis is sufficient to modify MASH pathology. NGM282 rapidly reduced liver fat and aminotransferase levels within 12 weeks [151], and improved fibrosis score, N‐terminal type III collagen propeptide, enhanced liver fibrosis (ELF) score, and other fibrosis‐related markers in an open‐label histological study, suggesting that FGF19‐like signaling may exert both antisteatotic and potentially antifibrotic effects [152]. Aldafermin further extended this mechanism into patients with F2‐F3 fibrosis and compensated cirrhosis. Although it did not consistently meet the primary histological endpoint, multiple secondary endpoints, including ELF, liver stiffness, and BA pharmacodynamic markers, improved [153, 154]. These findings suggest that the FGF19 pathway may be more closely aligned with fibrotic biology than with simple fat reduction, although its efficacy appears highly dependent on disease stage, endpoint selection, and treatment duration. In contrast, the ASBT inhibitor volixibat provides an important negative example. Although volixibat increased C4 and reduced cholesterol levels, confirming adequate target engagement, it failed to improve MRI‐PDFF, ALT, or histology, and diarrhea was common, ultimately leading to termination for lack of efficacy [155]. This indicates that simply altering BA enterohepatic flux is insufficient to reverse the complex lipotoxic‐inflammatory‐fibrotic network that drives MASH.

Therefore, the central value of BA‐targeted therapy in MASLD/MASH should not be reduced to lipid lowering or choleresis, but should instead be understood as remodeling of the gut‐liver metabolic and immune network. Clinical translation remains limited by short treatment durations, small sample sizes, reliance on surrogate endpoints, and insufficient evidence for histological benefit and long‐term clinical outcomes. Moreover, pruritus, lipid abnormalities, diarrhea, and safety concerns in advanced liver disease remain major barriers. Future studies should integrate BA profiles, gut microbiome signatures, fibrosis stage, and cardiovascular risk into a unified stratification framework to identify the patient populations most likely to benefit from BA signaling modulation.

### Emerging therapeutic applications of BAs

Beyond hepatobiliary diseases, recent studies have revealed the therapeutic potential of BAs and their derivatives in T2DM, neurodegenerative diseases, retinal degenerative disorders, and *C. difficile* infection, reflecting an expansion of BA‐based interventions from hepatobiliary indications toward systemic metabolic regulation and cytoprotection.

In T2DM, BAs regulate glucose homeostasis mainly through FXR, TGR5, and gut microbiota‐dependent mechanisms. OCA, an FXR agonist, improves insulin sensitivity and ameliorates markers of hepatic inflammation and fibrosis; however, its effects on the lipid profile suggest that systemic FXR activation should be approached with caution [156]. By contrast, TGR5‐related pathways are more directly linked to the incretin axis. Glycoursodeoxycholic acid reshapes the gut microbiota and BA pool, increases taurolithocholic acid levels and *Bacteroides vulgatus* abundance, thereby activating adipose TGR5 and promoting white adipose tissue thermogenesis [157]. TUDCA has been identified as a key effector mediating the insulin‐sensitizing effect of metformin in obese mice, improving insulin signaling through the nuclear factor erythroid 2‐related factor 2 antioxidant pathway [158]. Hyocholic acid species and 6α‐hydroxylated BAs further support this concept, as they improve glucose metabolism by activating TGR5, inhibiting FXR, and promoting GLP‐1 secretion [159, 160]. CA7S, which is increased after bariatric surgery, acts as a gut‐restricted TGR5 agonist that induces GLP‐1 release and improves glucose clearance while potentially avoiding gallbladder‐related adverse effects associated with systemic TGR5 activation, providing an important paradigm for the development of locally acting intestinal BA mimetics [121].

In neurodegenerative diseases, the major therapeutic value of UDCA/TUDCA lies more in cytoprotection than in canonical BA receptor signaling. Studies in Parkinson's disease and Alzheimer's disease suggest that UDCA improves mitochondrial function, reduces oxidative stress, inhibits apoptosis and neuroinflammation, and promotes autophagic clearance of abnormal protein aggregates [161]. In a phase II randomized trial in Parkinson's disease, high‐dose UDCA showed good safety and tolerability, and 31P‐MRS indicated target engagement in midbrain energy metabolism; however, subjective motor scores did not demonstrate a clear clinical benefit [162]. In amyotrophic lateral sclerosis, TUDCA treatment was associated with longer survival in a real‐world cohort, particularly among patients receiving higher doses, although this evidence remains limited by the retrospective design and potential selection bias [163]. Notably, insufficient delivery of UDCA to the brain remains a key obstacle. Mitochondria‐targeted nanovesicles loaded with UDCA can cross the BBB and improve mitochondrial function in Parkinson's disease models, suggesting that optimized delivery systems may determine the future translational value of UDCA‐based neuroprotection [164].

Retinal degenerative diseases provide a tissue context that is closer to clinical translation for the neuroprotective effects of UDCA/TUDCA. TUDCA protects retinal pigment epithelial cells from oxidative injury and endoplasmic reticulum stress by reducing reactive oxygen species, malondialdehyde, C/EBP homologous protein, and inflammatory mediators [165]. In an optic nerve crush model, TUDCA preserves retinal ganglion cells, maintains pattern electroretinography function, and suppresses Müller glial activation and inflammatory cytokine expression [166]. In a diabetic retinopathy model, early TUDCA treatment preserves retinal and visual function, suggesting that it may be more suitable as an early neuroprotective intervention rather than as a rescue therapy for late‐stage vascular complications [167]. Oral UDCA can enter the eye when the blood‐retinal barrier is disrupted and suppress photoreceptor death in an ex vivo retinal detachment model, further supporting its potential as an adjuvant therapy after retinal injury or surgery and in degenerative retinal diseases [168].

In recurrent *C. difficile* infection, the therapeutic logic of BAs shifts toward ecological restoration. Antibiotic exposure disrupts bacterial communities with BSH and 7α‐dehydroxylation capacity, leading to the accumulation of TCA, which promotes spore germination, and depletion of secondary BAs that inhibit *C. difficile* growth [169]. Observational data suggest that UDCA use in high‐risk patients is associated with a lower recurrence rate, although controlled trials are still required [170]. More mechanistic studies indicate that the efficacy of fecal microbiota transplantation is closely linked to restoration of BSH function; engineered or naturally BSH‐positive bacteria can reduce TCA‐mediated spore germination and lower *C. difficile* burden in mice [171, 172].

Overall, the essence of BA‐based therapy is not the simple supplementation of a single BA species, but the context‐dependent recalibration of homeostasis across multiple biological layers. Future strategies with genuine translational potential may include the development of tissue‐restricted BA receptor agonists, targeted delivery of UDCA/TUDCA, and precision interventions guided by BA profiles and microbiome functional signatures.

From a One Health perspective, the therapeutic application of BAs in human health extends beyond individual patient benefit and carries broader implications for public health and environmental sustainability. By targeting cholestatic liver diseases, MASLD, T2DM, and neurodegenerative disorders, BA‐based therapies may help reduce the long‐term burden of chronic metabolic diseases and limit unnecessary exposure to broad pharmacological interventions. Moreover, the development of gut‐restricted BA agonists, FXR modulators, and TGR5‐targeted strategies aligns with precision medicine. Thus, positioning BAs as key molecular hubs within the One Health framework may not only advance human therapeutic strategies but also contribute to a healthier and more sustainable future for humans, animals, and shared ecosystems.

## 6 APPLICATIONS OF BAS IN ANIMAL HEALTH

Similar to humans, animals raised under intensive production systems are highly susceptible to disorders such as dysregulated glucose and lipid metabolism and impaired intestinal barrier function [173, 174]. Traditionally, these conditions have been managed primarily through the use of antibiotics or other pharmaceuticals; however, such interventions inevitably exacerbate the problems of drug residues in ecosystems and the crisis of antimicrobial resistance. As potent regulators of metabolism and immunity, BAs have shown considerable potential to serve as alternatives to conventional intervention strategies and have therefore attracted increasing attention in the field of animal science. Table 1 summarizes the current applications of BAs as feed additives in monogastric and ruminant animals.

**Table 1:** Applications of BAs in animal health.

Animal types	BA types	Dose and duration	Functions	References
*Monogastric animals*				
IUGR pigs	HCA	0.4 g/kg diet, 28 days	Improve colonic development, intestinal barrier integrity, BA metabolism, gut microbiota, and immune homeostasis	[175]
	HDCA			
	CDCA			
Weaning piglets	HCA	0.06–2 g/kg diet, 7–42 days	Improve growth performance, nutrient utilization, intestinal barrier integrity, gut microbiota, antioxidant capacity, and immune‐endocrine function.	[176, 177, 178]
	HDCA			
	CDCA			
Finishing pigs	LCA	0.5 g/kg diet, 28‐112 days	Regulate BA profiles, systemic metabolism, feed efficiency, and backfat deposition, with HDCA reducing and LCA increasing lipid deposition.	[179, 180]
	HCA			
	CDCA			
	HDCA			
	TDCA			
	TUDCA			
*Poultry*				
Broiler chickens	HCA	0.06–1.5 g/kg diet or gavage administration, 14–42 days	Reduce hepatic lipid deposition, rebalance lipid and BA metabolism, remodel cecal microbiota, and improve antioxidant, immune, intestinal barrier, and detoxification functions.	[181, 182, 183, 184, 185]
	DCA			
	CDCA			
	HDCA			
	TCDCA			
Laying hens	HCA	0.03–0.24 g/kg diet, 28–168 days	Improve laying performance, lipid metabolism, cecal microbiota, immune function, and heat‐stress resistance	[186, 187]
	HDCA			
	CDCA			
Duck	HCA	0.25 g/kg diet, 30 days	Reduce abdominal fat deposition, enhance breast muscle growth, and improve energy allocation in ducks.	[188]
	HDCA			
	CDCA			
*Aquatic animals*				
Pacific white shrimp, tilapia, largemouth bass, and other aquatic species	HCA	0.016–0.5 g/kg diet, 32–105 days	Improve growth, lipid and BA metabolism, liver and gut health, immunity, gut microbiota, and muscle quality in aquatic animals.	[189, 190, 191, 192, 193, 194, 195]
	HDCA			
	CDCA			
*Ruminants*				
Meat‐producing ruminants	HCA	0.1–2.3 g/kg diet or DM,	Improve fat partitioning, meat quality, muscle oxidative metabolism, and rumen microbial function.	[196, 197, 198]
	HDCA	63–70 days		
	CDCA			
Lactating dairy cows	HCA	6–18 g/head/day, 84 days	Improve milk quality by regulating redox status and lipid metabolism.	[199]
	HDCA			
	CDCA			
Dairy goats	HCA	3–4 g/head/day, 35–56 days	Regulate milk fatty acid composition and enhance milk fat and plasma lipid metabolism, with stronger effects under fat‐supplemented diets.	[200, 201]
	HDCA			
	CDCA			
Transition dairy cows	HCA	10–20 g/head/day, 42 days	Improve energy adaptation, reduce β‐hydroxybutyrate, and alleviate inflammation and oxidative stress in transition dairy cows.	[174, 202]
	HDCA			
	CDCA			

*Note*: Literature search strategy for Table 1: Studies were identified from PubMed, Web of Science, and Google Scholar up to June 2026 using “Bile acid*” combined with “Supplementation” or “Additive*” and animal‐specific terms for pigs, poultry, aquatic animals, and ruminants. Specific terms included “IUGR pigs,” “Weaning piglets,” “Finishing pigs,” “Broiler chickens,” “Laying hens,” “Ducks,” “Pacific white shrimp,” “Tilapia,” “Largemouth bass,” “Meat‐producing ruminants,” “Lactating dairy cows,” “Dairy goats,” and “Transition dairy cows.” Original in vivo studies were included if they reported BA type, dose, duration, animal category, and production‐ or health‐related outcomes; reviews, conference abstracts, in vitro studies, non‐supplementation studies, and studies lacking clear supplementation information were excluded. Doses are presented as reported in the original studies. Dairy cows and dairy goats were generally supplemented on a grams‐per‐head‐per‐day basis, whereas most other animals were supplemented according to dietary concentration. Gavage doses are listed separately, and actual daily BA intake from dietary supplementation depended on feed intake. Durations indicate the BA supplementation periods reported in the original studies.

Abbreviations: BA, Bile acid; CDCA, Chenodeoxycholic acid; DCA, Deoxycholic acid; HCA, Hyocholic acid; HDCA, Hyodeoxycholic acid; IUGR, Intrauterine growth restriction; LCA, Lithocholic acid; TCDCA, Taurochenodeoxycholic acid; TDCA, Taurodeoxycholic acid; TUDCA, Tauroursodeoxycholic acid.

### Monogastric animal health

The weaning phase represents the most responsive window for BA nutritional intervention. Due to insufficient endogenous bile secretion and immature fat digestion capacity, exogenous porcine BAs can improve the apparent digestibility of crude fat and energy, thereby enhancing daily weight gain and antioxidant status in weaned piglets, indicating that their production‐enhancing effects are primarily mediated through improved nutrient utilization efficiency [176]. CDCA further increases the villus/crypt ratio, goblet cell count, and the expression of Zonula Occludens‐1, Occludin, and Claudin‐1, accompanied by an increase in *Prevotella*‐related bacteria, a decrease in *Dorea*, and remodeling of the serum metabolomic profile, suggesting that its effects extend to the barrier‐microbiota‐metabolite axis [177]. HDCA, in turn, tends to modulate intestinal endocrine and immune functions: in weaned piglets, it alters markers of enteroendocrine cell differentiation and GLP‐2 levels, while increasing globulin and IgA, indicating a potential influence on weaning adaptation through the enteroendocrine‐humoral immunity axis [178].

In low birth weight (LBW) or intrauterine growth‐restricted (IUGR) pigs, BA metabolic dysregulation appears to be an integral part of the disease phenotype rather than a mere epiphenomenon: IUGR alters hepatic BA synthesis, FXR/TGR5‐related signaling, and the colonic BA derivative profile, and these changes correlate with the abundance of specific microbial taxa [203]. Supplementation with a composite BA mixture targeting this deficiency improves colonic development and epithelial integrity, reduces d‐lactate, endotoxin, and diamine oxidase, and remodels the Firmicutes/Bacteroidetes ratio and colonic metabolic pathways [175]. Studies in LBW piglets further link “microbiota alteration—BA metabolic dysregulation—intestinal inflammation”: gut microbiota from LBW piglets can induce colonic inflammation in recipient mice, whereas supplementation with UDCA suppresses NF‐κB‐mediated inflammatory responses via FXR signaling and promotes M2 macrophage polarization, thereby restoring a degree of intestinal immune homeostasis [204]. This finding suggests that BAs may serve as key intermediary molecules connecting gut microbiota dysbiosis and mucosal immune imbalance.

In growing‐finishing pigs, supplementation with a composite BA mixture does not significantly alter fecal microbiota composition, but does increase levels of secondary BAs such as HDCA and affects multiple serum metabolic pathways related to energy and lipid metabolism [179]. This suggests that, in adult pigs, BAs may act primarily by modulating the BA profile and systemic metabolic status, rather than by directly relying on structural changes in the gut microbiota or markedly promoting growth. Importantly, different BA molecules exert distinct—and even opposing—physiological effects in pigs. HDCA promotes TGR5/hormone‐sensitive lipase‐related lipolysis and tends to reduce backfat thickness, whereas LCA promotes PPARγ‐, SREBP‐1c‐, and fatty acid synthase‐related lipid synthesis [180]. Therefore, future applications should shift from “total BA supplementation” to precision nutritional regulation based on BA profile, developmental stage, and health status.

### Poultry health

The decline in laying performance in hens is often accompanied by lipid homeostasis imbalance, hepatic lipid accumulation, and increased oxidative stress, reflecting a compromised capacity for lipid synthesis, transport, and clearance during the late stages of high‐intensity egg production. In late‐laying hens, porcine BAs reduce serum TG, total cholesterol, and LDL‐C, decrease abdominal fat deposition, and enrich beneficial bacteria such as *Lactobacillus* and *Bifidobacterium* [186]. These changes shift cecal function toward enhanced FA degradation, BA biotransformation, and SCFA metabolism, indicating that the core mechanism by which BAs improve laying performance is not merely a simple increase in digestibility, but rather the alleviation of lipid metabolic congestion under the background of aging and sustained egg production. Similarly, in broilers fed a HFD, BA supplementation reduces plasma and hepatic TG levels and alleviates hepatic lipid deposition, accompanied by reduced hepatic fatty acid synthase expression, altered hepatic CA, CDCA, and non‐12‐OH/12‐OH BA profiles, and remodeling of cecal microbiota, including *Bacteroides*, *Bifidobacterium*, *Escherichia*, and *Lactobacillus* [181]. These findings further demonstrate that BAs mitigate HFD‐induced hepatic lipid deposition primarily through a pathway of “microbiota remodeling—BA pool reshaping—hepatic lipid metabolism rebalancing.”

Notably, the regulation of lipid deposition by BAs is not a simple “lipid‐lowering effect,” but rather resembles an energy allocation modulation that depends on molecular structure and physiological context. In Zhijiang ducks, BA supplementation simultaneously reduces abdominal fat, increases breast muscle weight and myofiber cross‐sectional area, and upregulates signaling pathways related to *NR1H4*/FXR, *PPARα*, carnitine palmitoyltransferase 1α, and insulin‐like growth factor 1/2, suggesting that BAs may redirect excess energy from fat storage toward muscle deposition by enhancing lipid oxidation [188]. However, this effect is not universal. When administered alone to broilers, taurochenodeoxycholic acid instead promotes abdominal fat deposition, upregulates *SREBP‐1*, CCAAT/enhancer‐binding protein α, and *ELOVL6*, and suppresses adipose triglyceride lipase and carnitine palmitoyltransferase 1, indicating that the beneficial effects of BAs are not universally generalizable; the ultimate phenotype depends on BA type, dosage, host species, dietary composition, and the reshaping of the BA pool by the gut microbiota [182].

In models of heat stress and disease, the actions of BAs extend beyond lipid metabolism to immunometabolic regulation. Supplementation with 120 mg/kg BAs in heat‐stressed laying hens reduces corticosterone, heat shock protein 70, and IL‐1β, increases IgA and IgM levels, improves laying performance, and links arachidonic acid‐related metabolites to immune transcriptional networks, including major histocompatibility complex class I, interleukin‐2 receptor, and transforming growth factor‐β receptor, suggesting that BAs may reprogram immune responses via lipid mediators [187]. In models of *Salmonella* infection, necrotic enteritis, and LPS challenge, BAs act respectively by enhancing the mucus layer/MUC2 barrier, reducing TLR4/TLR5‐IL‐1β inflammatory signaling, restoring the ileal BA pool, and activating the FXR/LXRα‐mediated hepatobiliary detoxification pathway to promote LPS clearance [183, 184, 185].

### Aquatic animal health

The replacement of fishmeal with plant‐based diets reduces the supply of cholesterol, taurine, and endogenous/exogenous BAs, which readily leads to BA pool insufficiency, limited lipid digestion, and increased hepatic metabolic load. In yellowtail kingfish, taurocholate enters the host BA pool, improves fat digestibility, and feedback‐inhibits BA synthesis, demonstrating that exogenous BAs can directly reshape enterohepatic circulation efficiency [189]. Studies in tilapia and spotted seabass further show that combined supplementation of cholesterol with BAs, or a cholesterol‐taurine‐glycine mixture, activates the FXR‐FGF19/BSEP‐related BA transport and feedback pathways, reduces ALT/AST activities, alleviates hepatocyte vacuolization and fatty liver, and enhances BA pool hydrophilicity and metabolic safety by reducing BSH‐producing bacteria and increasing the proportion of conjugated CDCA [190, 205].

In crustaceans, which lack or have a limited capacity for BA synthesis, this axis remains valid. In Pacific white shrimp, appropriate levels of dietary BAs improve growth, lipase and protease activities, high‐density lipoprotein levels, antioxidant capacity, and NF‐κB‐mediated antimicrobial peptide expression, while also remodeling the gut microbiota and its BA‐transforming functions. BAs can partially replace dietary cholesterol requirements, mitigating the growth and microbiota imbalances caused by low‐cholesterol plant‐based diets [191, 192]. In Chinese mitten crabs, cholesterol provides the BA precursor, and taurine facilitates its conversion and turnover; their synergy expands the hepatopancreatic BA pool, improves lipid metabolism, and enhances mTOR nutrient sensing. Moreover, the methionine‐taurine interaction suggests that sulfur amino acid metabolism is a critical upstream limiting factor for BA conjugation capacity [193, 206].

Notably, the effects of BAs exhibit a clear dose window. In largemouth bass, 150‐300 mg/kg BAs improve chemical, physical, and immunological barriers, and germ‐free zebrafish experiments validate both direct and microbiota‐mediated effects; however, higher doses can suppress some immune barrier genes [194]. In white shrimp, 0.5 g/kg BAs also pose a risk of oxidative damage, indicating that application requires precise quantification based on species, BA composition, and feed matrix [192]. On the other hand, studies in abalone extend the functions of BAs to product quality: appropriate supplementation increases muscle hardness and water‐holding capacity through the p38/MAPK pathway, collagen maturation, AKT/mTOR‐mediated protein deposition, glycolysis inhibition, and the antioxidant network [195]. These findings demonstrate that BAs can serve as dual regulators of “health‐quality,” extending beyond liver protection and digestion enhancement.

### Ruminant health

Recent studies indicate that BAs in ruminants are not merely lipid emulsifiers, but endogenous signaling nodes that connect gut microbiota, hepatic energy metabolism, milk and meat quality, and inflammatory homeostasis.

Studies in transition dairy cows particularly suggest that BAs are not simply passive byproducts of metabolic disturbance, but may participate in the adaptation to negative energy balance. During the periparturient period, large amounts of non‐esterified fatty acids are mobilized and delivered to the liver, where their partitioning among complete oxidation, re‐esterification, and ketogenesis directly determines hepatic metabolic burden and health status [2]. In this process, the BA profile undergoes marked remodeling. CDCA, CA, and their derivatives may act as key hubs linking gut microbiota with host metabolic indicators, including glucose, TG, non‐esterified fatty acids, and β‐hydroxybutyrate [207]. These findings suggest that the “BA‐gut microbiota‐hepatic lipid load‐energy adaptation” axis may represent an important mechanism underlying periparturient metabolic homeostasis.

Exogenous BA supplementation further supports this concept. Its significance extends beyond increasing milk fat percentage or reducing β‐hydroxybutyrate concentrations in periparturient cows; rather, it suggests that reshaping the BA pool and microbial BA‐transforming capacity may redirect lipid metabolic flux in dairy cows [174]. In transition cows, BA supplementation enriches BA‐transforming bacteria and enhances functional pathways related to BSH, 7α‐hydroxysteroid dehydrogenase, and BA‐inducible protein E, implying that secondary BAs generated by hindgut microbiota may serve as important signals regulating hepatic FA oxidation [174]. Studies using UDCA and bile salts also show that BA intervention can improve ruminal volatile fatty acid production, reshape ruminal and fecal microbiota, and alleviate inflammation and oxidative stress [202]. These observations suggest that the effects of BAs extend beyond a single process of nutrient absorption and more closely resemble systemic regulation of metabolic homeostasis. This perspective is also applicable to lactating ruminants. Studies in mid‐lactation dairy cows collectively indicate that BAs do not simply “enhance fat absorption,” but may influence milk quality by modulating redox status, circulating lipid metabolism, and the availability of FA substrates for mammary lipid synthesis [199].

Studies in dairy goats further highlight the importance of dietary context. Under starch‐rich dietary conditions, BA supplementation alters milk FA composition, hindgut BA profiles, and rectal microbial function, indicating that milk fat synthesis in ruminants should not be interpreted solely from the perspective of rumen fermentation; small intestinal absorption and hindgut BA transformation should also be incorporated into the mechanistic framework [200]. Under fat‐supplemented dietary conditions, the effects of BAs on milk fat and plasma lipid metabolism appear more pronounced, suggesting that BA application may be more suitable when combined with specific fat sources or high‐energy diets, rather than being used as a universal additive across all dietary conditions [201]. Collectively, these findings indicate that mammary lipid synthesis is not determined solely by dietary fat supply, but is jointly influenced by a continuum of processes involving the intestinal BA pool, microbial transformation capacity, circulating lipid substrates, and mammary uptake and utilization.

Research in meat‐producing ruminants further extends the functions of BAs to fat partitioning, rumen ecology, and skeletal muscle energy metabolism. In lambs fed high‐energy diets, BA supplementation reduces tail and subcutaneous fat deposition without necessarily compromising intramuscular fat, suggesting that BAs may participate in regulating fat distribution rather than simply suppressing adipogenesis [196]. In Hu sheep, supplementation with rumen‐protected BAs increases the proportion of type I oxidative muscle fibers, enhances oxidative phosphorylation, suppresses glycolysis, and reduces drip loss [197]. These findings suggest that circulating BAs or their metabolites may contribute to meat quality formation by reshaping the energy phenotype of skeletal muscle. Studies in culled ewes further show that BAs not only affect nutrient digestibility and hepatic function, but are also associated with rumen microbial quorum sensing and biofilm formation [198]. This suggests that the improvement in feed efficiency may not merely result from changes in microbial composition, but may also involve enhanced cooperative degradation by microorganisms attached to feed particles, providing a new perspective for understanding feed utilization through rumen microbial synergistic digestion.

From a One Health perspective, BA‐based nutritional intervention is not only a technical strategy for improving metabolic health and milk and meat quality in animals, but is also relevant to human nutrition supply, animal welfare, and the sustainability of livestock production. By reducing the risk of metabolic disorders, improving feed utilization efficiency, and decreasing resource consumption, BAs may be integrated into the shared “human‐animal‐environment” health framework, providing a new entry point for precision nutritional regulation and sustainable livestock production.

## 7 CONCLUSIONS AND FUTURE PERSPECTIVES

This review systematically summarized the synthesis, enterohepatic circulation, microbial transformation, and extrahepatic metabolism of BAs, with particular emphasis on their roles in regulating glucose and lipid metabolism, intestinal barrier integrity, immune homeostasis, and T cell differentiation through the activation of receptors such as FXR and TGR5, as well as through microbiota‐derived secondary or modified BAs. We further discussed how host physiological status, circadian rhythm, diet, behavior, environmental exposure, and surgical interventions reshape the BA pool and thereby influence host health. In human health, BA‐related interventions have shown considerable translational potential in cholestatic liver diseases, MASLD, T2DM, neurodegenerative diseases, retinal degenerative disorders, and intestinal infections. In animal health, BAs, as non‐antibiotic nutritional modulators, can improve lipid utilization, intestinal health, immune function, stress resistance, and production performance. They may also support food safety at the source and ecological sustainability by reducing dependence on pharmacological interventions, lowering the risk of drug residues, and alleviating antimicrobial resistance pressure. Therefore, BAs are not only key signaling molecules linking host metabolism with microbial functions, but also provide a new perspective for integrating human health, animal health, and ecosystem health.

However, several key challenges remain before BA research can move from mechanistic understanding to practical application (Figure 4). First, the development of BA derivatives and receptor‐targeted molecules will be an important direction for advancing the translational potential of this field. Natural BAs exhibit structural diversity and broad receptor activity, but their systemic effects may also lead to insufficient targeting, limited tissue specificity, and adverse reactions. Future studies should focus on developing BA derivatives with improved receptor selectivity and metabolic stability, such as gut‐restricted TGR5 agonists, selective FXR modulators, low‐toxicity UDCA/TUDCA derivatives, and novel molecules that mimic functional microbiota‐derived BAs. Meanwhile, it is necessary to further elucidate the affinity, signaling bias, and tissue‐specific effects of different BA structures toward receptors such as FXR, TGR5, pregnane X receptor, and vitamin D receptor, thereby avoiding oversimplification of BA signaling as the action of a single receptor or pathway. For animal production, the development of safe, stable, and scalable BA complexes or functional derivatives will also be critical for improving their industrial application.

**Figure 4:**
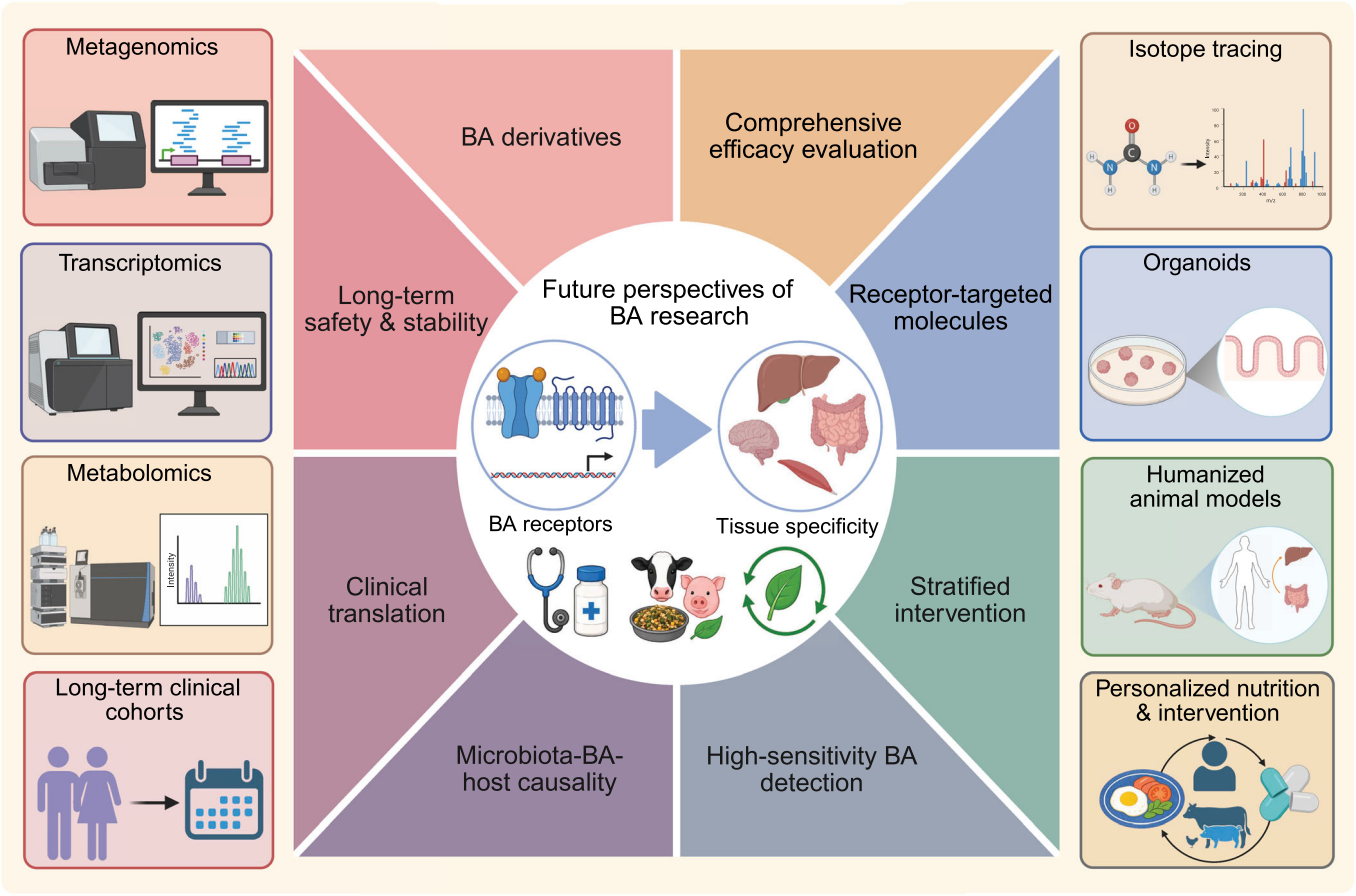
Future perspectives of bile acid (BA) research. Future studies on BAs should advance from mechanistic characterization toward precision translation by integrating BA derivatives, receptor‐targeted molecules, stratified intervention, high‐sensitivity BA detection, and clinical translation. Multi‐omics, organoids, isotope tracing, humanized animal models, and long‐term clinical cohorts will help clarify BA‐microbiota‐host causality, identify responders and non‐responders, and support personalized nutrition, therapeutic development, and sustainable animal production.

Second, precision nutrition represents an important pathway for translating BA research into human health management and animal health production. Diet, circadian rhythm, host physiological status, gut microbiota composition, and environmental exposure can all reshape the BA profile and determine the biological outcomes of BA signaling. This may explain why the same dietary pattern or BA intervention produces divergent, or even opposite, effects across different individuals, species, and application contexts. Future precision nutrition strategies should integrate BA profiling, microbiome functional features, host metabolic indicators, and dietary information to identify responders and non‐responders. In humans, this approach may facilitate the development of personalized dietary or pharmacological interventions for obesity, diabetes, MASLD, cholestatic diseases, and inflammatory disorders. In animals, BA application should shift from uniform supplementation toward stratified intervention, matching BA type, dosage, and formulation with species‐specific BA metabolism, diet composition, production stage, health status, and production goals. For example, young animals may benefit from BA interventions that improve intestinal maturation, nutrient absorption, and immune resilience, whereas high‐producing dairy cows, laying hens, and aquaculture species may require BA strategies targeting lipid partitioning, redox balance, hepatic protection, product quality, and feed efficiency. Meanwhile, evaluation criteria should move beyond production performance alone and incorporate metabolic status, intestinal health, immune homeostasis, reduced drug use, environmental benefits, dose windows, long‐term safety, and cost‐effectiveness. Such precision BA nutrition strategies may help reduce antibiotic dependence, improve resource utilization, and promote more sustainable animal production.

Finally, clinical translation remains a central bottleneck for BA‐targeted strategies. Although FXR agonists, FGF19 analogs, IBAT inhibitors, UDCA/TUDCA, and other BA‐related agents have shown potential in various diseases, their clinical application is still limited by heterogeneous efficacy, adverse reactions, long‐term safety concerns, and endpoint selection. For example, pruritus, lipid profile alterations, and safety concerns in advanced liver disease associated with FXR agonists; diarrhea associated with IBAT inhibitors; insufficient delivery efficiency of UDCA/TUDCA in neurological diseases; and interindividual differences in BA profiles and gut microbiota all restrict the stable translation of therapeutic effects. Future clinical studies should strengthen long‐term randomized controlled trials and integrate BA profiles, gut microbial functions, disease stage, fibrosis severity, and cardiovascular risk to establish more precise patient stratification and efficacy prediction systems. In addition, BA detection methods need to advance toward higher sensitivity, higher throughput, lower cost, and greater clinical feasibility, thereby promoting their application as biomarkers for disease screening, therapeutic monitoring, and prognostic evaluation.

Overall, future BA research should move beyond research models centered on a single molecule, disease, or species, and instead adopt cross‐species, cross‐system, and interdisciplinary approaches. By integrating isotope tracing, organoids, germ‐free or humanized animal models, long‐term clinical cohorts, and multi‐omics technologies such as metabolomics, metagenomics, and transcriptomics, future studies may more accurately reveal the causal relationships among BAs, microbiota, receptors, and hosts in different contexts. These efforts will promote the development of BA‐based precision nutrition strategies, innovative therapies, and next‐generation BA derivatives, thereby improving human health, enhancing animal production, and reducing environmental pressure. Therefore, BAs are not merely metabolic regulators, but also represent a promising translational platform linking human, animal, and environmental health, opening new avenues for integrated One Health solutions.

## AUTHOR CONTRIBUTIONS

**Lei Li**: conceptualization; writing–original draft; writing—review and editing. **Shuai Liu**: supervision; writing—review and editing. **Zhijun Cao**: funding acquisition; writing—review and editing. **Yangchun Cao, Shengru Wu**, and **Lu Deng**: writing—review and editing. **Junhu Yao**: supervision; conceptualization; funding acquisition; writing—review and editing. **Jun Zhang**: supervision; conceptualization; funding acquisition; writing—review and editing. All authors have read the final manuscript and approved it for publication.

## CONFLICT OF INTEREST STATEMENT

The authors declare no conflicts of interest.

## ETHICS STATEMENT

No animals or humans were involved in this study.

## Data Availability

No new data have been generated in this study. Supplementary materials (graphical abstract, slides, videos, Chinese translated version and update materials) may be found in the online DOI or iMetaOmics http://www.imeta.science/imetaomics/.
